# Molecular Insights into O-Linked Sialoglycans Recognition by the Siglec-Like SLBR-N (SLBR_UB10712_) of *Streptococcus gordonii*

**DOI:** 10.1021/acscentsci.3c01598

**Published:** 2024-02-07

**Authors:** Cristina Di Carluccio, Linda Cerofolini, Miguel Moreira, Frédéric Rosu, Luis Padilla-Cortés, Giulia Roxana Gheorghita, Zhuojia Xu, Abhishek Santra, Hai Yu, Shinji Yokoyama, Taylor E. Gray, Chris D. St. Laurent, Yoshiyuki Manabe, Xi Chen, Koichi Fukase, Matthew S. Macauley, Antonio Molinaro, Tiehai Li, Barbara A. Bensing, Roberta Marchetti, Valérie Gabelica, Marco Fragai, Alba Silipo

**Affiliations:** † Department of Chemical Sciences, University of Naples Federico II, Naples 80138, Italy; ‡ Magnetic Resonance Centre (CERM), CIRMMP, and Department of Chemistry “Ugo Schiff”, University of Florence, Sesto Fiorentino 50019, Italy; § IECB Institut Européen de Chimie et Biologie, Pessac 33600, France; ∥ Giotto Biotech s.r.l., Sesto Fiorentino 50019, Italy; ⊥ Shanghai Institute of Materia Medica, Chinese Academy of Sciences, Shanghai 201203, China; # Department of Chemistry, University of California, Davis, Davis, California 95616, United States; ∇ Graduate School of Science, Osaka University, Osaka 565-0871, Japan; ■ San Francisco Veterans Affairs Medical Center and University of California, San Francisco, California 94121, United States; ○ Department of Chemistry, University of Alberta, Edmonton, Alberta T6G 2R3, Canada; ▲ Department of Medical Microbiology and Immunology, University of Alberta, Edmonton, Alberta T6G 2R3, Canada

## Abstract

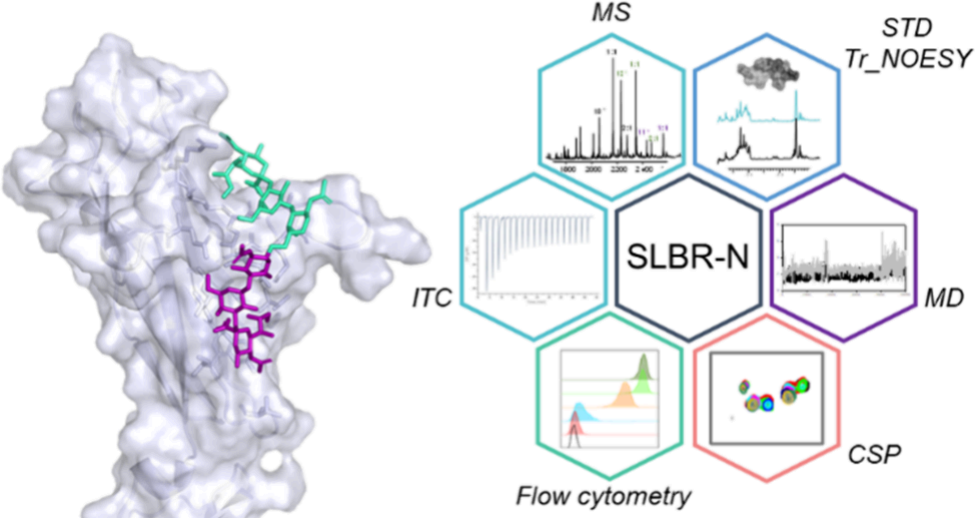

*Streptococcus gordonii* is a Gram-positive bacterial species that typically colonizes the human oral cavity, but can also cause local or systemic diseases. Serine-rich repeat (SRR) glycoproteins exposed on the *S. gordonii* bacterial surface bind to sialylated glycans on human salivary, plasma, and platelet glycoproteins, which may contribute to oral colonization as well as endocardial infections. Despite a conserved overall domain organization of SRR adhesins, the Siglec-like binding regions (SLBRs) are highly variable, affecting the recognition of a wide range of sialoglycans. SLBR-N from the SRR glycoprotein of *S. gordonii* UB10712 possesses the remarkable ability to recognize complex core 2 *O*-glycans. We here employed a multidisciplinary approach, including flow cytometry, native mass spectrometry, isothermal titration calorimetry, NMR spectroscopy from both protein and ligand perspectives, and computational methods, to investigate the ligand specificity and binding preferences of SLBR-N when interacting with mono- and disialylated core 2 *O*-glycans. We determined the means by which SLBR-N preferentially binds branched α2,3-disialylated core 2 *O*-glycans: a selected conformation of the 3′SLn branch is accommodated into the main binding site, driving the sTa branch to further interact with the protein. At the same time, SLBR-N assumes an open conformation of the CD loop of the glycan-binding pocket, allowing one to accommodate the entire complex core 2 *O*-glycan. These findings establish the basis for the generation of novel tools for the detection of specific complex *O*-glycan structures and pave the way for the design and development of potential therapeutics against streptococcal infections.

## Abstract 2

A multidisciplinary approach combining NMR spectroscopy, native mass spectrometry, flow cytometry, ITC and MD simulation unveiled the binding details of core 2 *O*-glycans to the Siglec-like adhesin SLBR-N of *S. gordonii*.

## Introduction

The human oral cavity is densely populated with bacteria, some of which can be associated with a variety of systemic infections.^1^ Bacteremia may result from lesions in the oral epithelium, occurring for instance after dental manipulation, and also following daily procedures, such as chewing, toothbrushing, and flossing. Once in the bloodstream, bacteria can reach and colonize other organs and consequently establish infection and inflammation. Streptococcal bloodstream infections are among the most frequent causes of infective endocarditis (IE), an inflammatory disease that impacts the endocardium and causes serious damage of the heart.^2^ In particular, *Streptococcus sanguinis*, *Streptococcus gordonii*, and *Streptococcus mitis/oralis*, commensals of the oral microbiota, are common in patients affected by IE.^3^ Despite the guidelines on prevention, diagnosis, and treatment,^4^ IE incidence has remained unchanged over the past 30 years. Antibiotic administration, the treatment of choice, often fails due to antibiotic resistance, thus making urgent the development of novel measures and approaches for IE prevention and treatment.

*S. sanguinis*, *S. gordonii*, and *S. oralis* express surface sialic acid-binding adhesins, including serine-rich repeat glycoproteins (SRRPs)^5^ and AsaA proteins^6^ that mediate adherence to host sialylated glycoproteins and tissue colonization. Generally, the structural organization of SRRPs includes an N-terminal signal peptide, a ligand binding region (BR) positioned between two SRR regions (a short SRR1 and long SRR2), and a C-terminal LPXTG motif anchored to the cell wall (Scheme S1). The “Siglec-like” BRs (SLBRs) include two domains that are crucial for glycan recognition: the Siglec domain and the adjacent Unique domain, involved in the modulation of the Siglec domain conformation. The SLBRs mediate recognition of α2–3-linked sialic acid-galactose epitopes located at the termini of O-linked glycans displayed on mucins and mucin-like proteins. The O-glycosylated host proteins bound by the SLBRS include the salivary mucin MUC7^7,8^ and platelet glycoprotein Ibα,^9,10^ and these interactions may contribute to oral colonization and IE, respectively.

*Streptococcus gordonii*, an oral commensal sometimes associated with periodontitis, can act as an opportunistic pathogen and cause a variety of systemic diseases, including empyema in the lung, spondylodiscitis in the spine, perihepatic abscess in the liver, and endocarditis.^11^

Despite the conserved structural fold of the Siglec domains and the crucial role of a ΦTRX motif in sialoglycan binding,^11,12^ the primary structures of SLBRs are highly divergent and show different selectivity for α2–3 sialoglycans. Recent chimeragenesis experiments pointed out how the specificity of Siglec-like adhesins for different ligands may be determined by hypervariability in the CD, EF, and FG loops of the V-set Ig fold in the Siglec domain, influencing the interaction with host cells, and then pathogenicity and commensalism.^13^ The diversity of ligands recognized is thought to parallel the range of *O*-glycan structures displayed on MUC7. Importantly, the SLBR ligand repertoire impacts the recognition of plasma and platelet glycoproteins, in addition to virulence and pathogenesis.^13,14^ Indeed, loop alterations in SLBRs modify the recognition of glycoforms of MUC7, revealing the importance of SLBR selectivity in bacterial adhesion to host receptors and then their potential implications for bacterial infections. The SLBRs of SRR adhesins from *Streptococcus gordonii* M99 and Challis strains, SLBR-B and SLBR-H, respectively, have been extensively characterized,^12−17^ and their contribution to pathogenicity was demonstrated in animal models of endocarditis. SLBR-B is selective for a core 1 trisaccharide known as sialyl T-antigen (sTa), whereas SLBR-H shows broader specificity and can bind sTa as well as the closely related structures 3′-sialyl-*N*-acetyllactosamine (3′SLn) and sialyl Lewis C (sLe^C^). In contrast, SLBR-N of *S. gordonii* strain UB_10712_ binds more avidly to different α2,3 sialoglycans, in particular to 3′-sialyl-*N*-acetyllactosamine (3′SLn) more avidly than to sialyl-T-antigen (sTa or) or sialyl Lewis C (sLe^C^). Despite that SLBR-H and SLBR-N share 80% sequence identity, only the latter recognizes fucosylated and sulfated derivatives of 3′SLn, including sialyl Lewis X (sLe^X^) and 6S-sLe^X^.^6^ Interestingly, SLBR-N also displayed the ability to bind larger, branched disialylated core 2 *O*-glycans, as demonstrated by probing glyco-engineered HEK293 cells and glycan arrays.^14,18^ However, the means by which SLBR-N may preferentially bind disialylated core 2 glycans is unclear. Furthermore, the ability of SLBR-N to detect O-linked sialoglycans on CD44, a complex transmembrane glycoprotein common marker of cancer stem cells (CSCs) in breast cancer, makes SLBR-N particularly attractive not only for its biological role, but also as a tool to detect core 2 disialylated *O*-glycans on tumor cells.^20^ In addition, SLBR-N has been recently shown to enhance the infectivity and transmissibility of HIV-1 by binding the *O*-glycans exposed on the surface of the envelope glycoprotein gp120 and increasing the affinity of HIV-1 for the CD4 receptor.^21^

To deeply understand how SLBRs can discriminate between closely related sialoglycan structures, and with the long-term goal of developing potential mimetics to hinder streptococcal infections, we undertook a comprehensive study of the SLBR-N recognition and binding to chemoenzymatically synthesized monosialylated and complex core 2 branched *O*-glycans.^19^ We employed a combination of multidisciplinary and complementary methods, consisting of flow cytometry, high-resolution ligand- and protein-based NMR experiments, native mass spectrometry, isothermal titration calorimetry, and computational approaches, including docking studies, molecular dynamic simulations, and CORCEMA-ST (complete relaxation and conformational exchange matrix analysis of saturation transfer), to achieve information about binding preferences, affinities, and 3D molecular features of such protein–ligand complexes. Our results showed a preference of SLBR-N in interacting with glycans containing a sialylactosamine branch; moreover, the presence of two sialic acids, as in disialylated core 2 *O*-glycan structures, results in a wider protein binding site, thus strengthening the interaction.

## Results

### SLBR-N Recognition of Host Glycans

To dissect the interaction of SLBR-N with host *O*-glycans, we analyzed the protein binding to host ligands. These include 3′-sialylactosamine (3′SLn, ligand **1**), sialyl-T-antigen (sTa-Thr, ligand **2**), and monosialylated core 2 *O*-glycans (ligands **3** and **4**); we then focused on the more complex disialylated core 2 *O*-glycans (ligands **5** and **6**, Scheme S2), containing both 3′SLn and sTa-Thr branches.

#### Flow Cytometry Analysis

**Figure 1:**
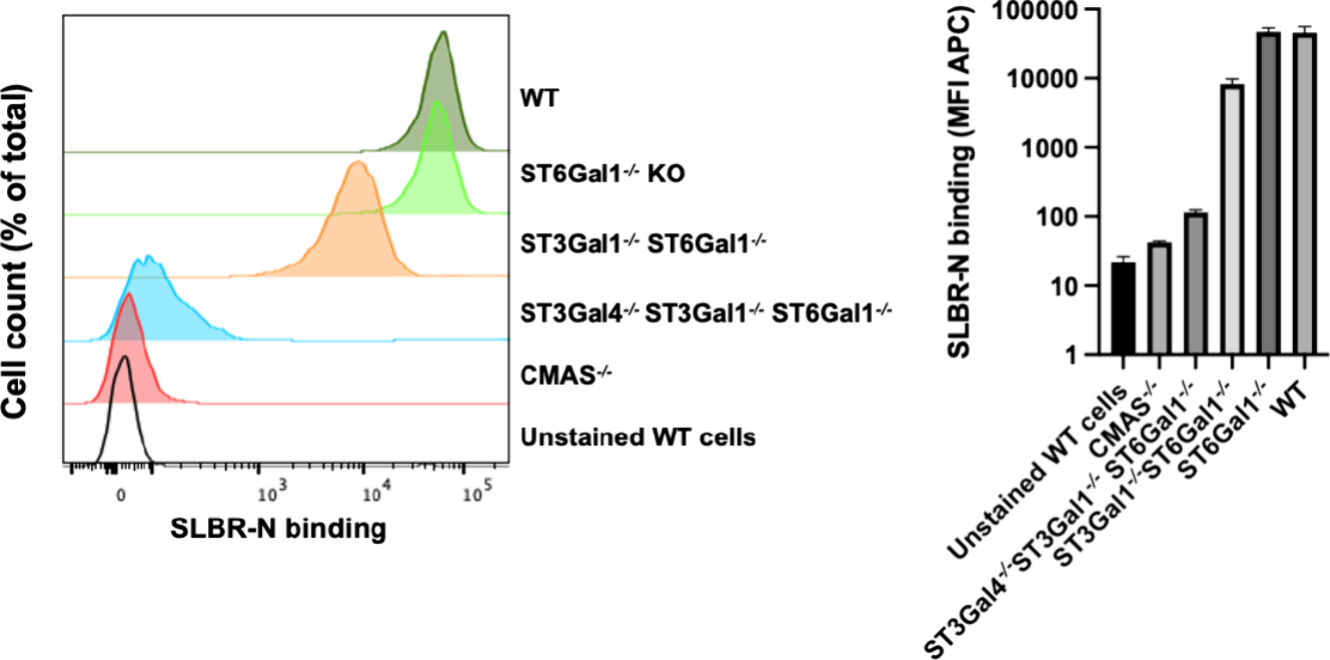
Flow cytometry analysis. SLBR-N binding to U937 WT (green), ST6Gal1 KO (lime green), ST3Gal1/ST6Gal1 KO (orange), ST3Gal1/4, ST6Gal1 KO (blue), CMAS KO (red), and unstained WT (black) cells. Left panel: Flow cytometry histograms. Right panel: Quantification of the mean fluorescence intensity (MFI) for three technical replicates.

The ability of SLBR-N to preferentially recognize α2–3-linked sialic acid containing glycans has been confirmed by flow cytometry experiments as follows. We knocked out several key sialyltransferases from U937 cells and tested the binding of fluorescently labeled SLBR-N to cells by flow cytometry (Figure 1). Knocking out ST6Gal1, which installs α2–6-linked sialic acid onto *N*-glycans, had no impact on SLBR-N binding. Further deletion of ST3Gal1, an enzyme that installs α2–3-linked sialic acid onto the core 1 branch of *O*-glycans (Scheme 2), led to the 4-fold decrease in binding of SLBR-N to cells. Further deletion of ST3Gal4, which likely installs α2–3-linked sialic acid onto the core 2 branch of *O*-glycans, caused a much greater impact on SLBR-N binding, decreasing binding by another 100-fold. It has previously been shown that both ST3Gal1 and ST3Gal4 are implicated in making the ligand for SLBR-N.^5,18,20^ This is supported by our findings that, however, point to ST3Gal4 as being the more critical enzyme in creating ligands recognized by SLBR-N on cells.

#### Native Mass Spectrometry and Binding Affinities

**Figure 2:**
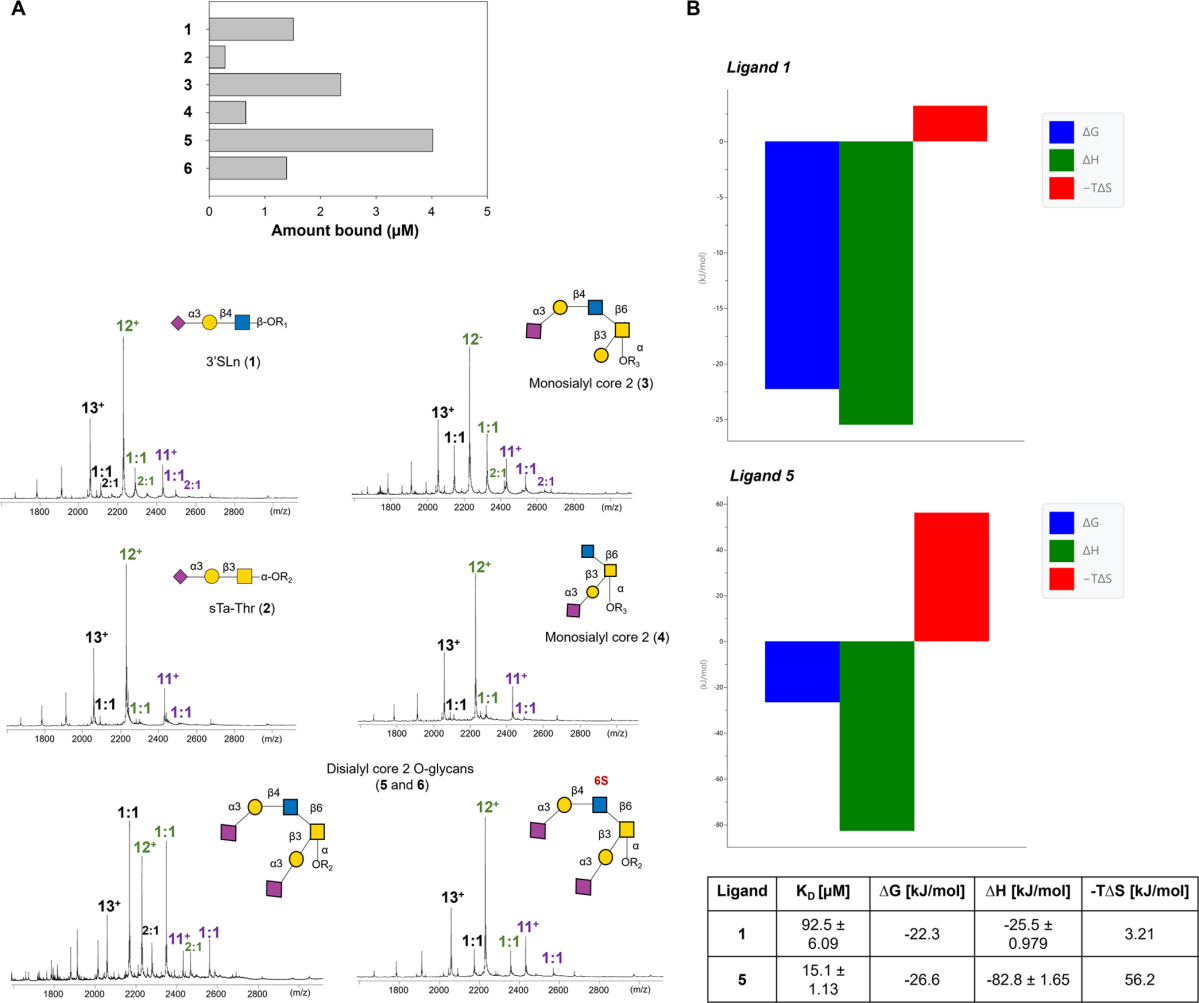
(A) Native mass spectrometry analysis. Electrospray mass spectra of protein SLBRN with ligands **1**–**6**. The 1:1 ligand:protein peaks were considered; lower intensity peaks corresponding to a 2:1 ligands:protein complex could be also observed for **1**, **4**, and **6**, likely due to nonspecific interactions. R1, ethanolamine; R2, threonine; and R3, methoxybenzene. The histogram derived from the MS-determined equilibrium association constants (see the Supporting Information) reflects the different affinity of SLBR-N for the different sialoglycans, revealing the following ligand preference: **5** ≫ **3** > **1**/**6** ≫ **4** > **2**. (B) Isothermal titration calorimetry analysis. Thermodynamic profiles for the interaction of **1** and **5** with SLBR-N, as measured by ITC experiments. Affinity constants and thermodynamic parameters of the interactions were shown in the table.

Native electrospray ionization mass spectrometry (ESI–MS) was used to obtain the binding affinities of the investigated glycans for SLBR-N. First, MS analysis on SLBR-N was performed using ion mobility MS.^21^ The charge states 12^+^ and 13^+^ were compact (Figure S1A); hence, presumably the protein was well folded and produced via a charged residue process and used for the quantification. Lower charge states (11^+^, 10^+^...) were not sufficiently declustered for quantification, while higher charge states could be partially unfolded, presumably produced partially by chain ejection, and, for this reason, were excluded from the quantification. Electrospray mass spectra then were acquired on the protein SLBR-N in complex with ligands **1**–**6** (Figure 2A). Stoichiometry and quantification of the complexes were then obtained from the position and the relative intensities of the peaks, respectively.^22−24^ Thus, ESI–MS allowed one to determine the global equilibrium binding constants (sum of 1:1 complexes and of 2:1 complexes) of the different systems (Figures 2A and S1A). Ligand **5** had the highest affinity for SLBR-N, with the stronger intensity of the peak corresponding to the 1:1 complex, followed by ligands **3** and **1** that also showed good binding, with the latter comparable to **6**. Regarding ligands **2** and **4**, the intensity was very low even at a 1:1 ligand:protein ratio, diagnostic of a weak affinity of SLBR-N for these ligands (*K*_D_ > 100 μM). The MS-derived affinity constants for these two ligands also serve to prove that the 1:1 and 2:1 complexes detected for the others are specific in the sense that they exist in solution and are not MS artifacts. However, as no NMR signal was found for a second binding site (see below), we conclude that the 2:1 complex detected by MS is not site-specific, but rather the sum of weakly interacting complexes over the protein surface.

#### Isothermal Titration Calorimetry

ITC was also performed to gain information regarding affinity constants (*K*_D_) and thermodynamic parameters of the binding for the two main ligands (**1** and **5**) (Figures 2B and S1B). In both cases, the formation of the complex was spontaneous (Δ*G* < 0), and the reaction was enthalpically driven, with Δ*H* more negative for ligand **5** with respect to **1** (Figure 2B and Supporting Information). The ITC results fitted well into a single-site binding model, and the derived *K*_D_ value agreed with those obtained by native MS (see below for further discussion on ITC results). Therefore, ESI–MS, ITC, and flow cytometry analyses provided information on the SLBR-N preferential recognition of 3-linked host sialoglycans and the binding affinities of protein–ligand complexes, showing a net preference of SLBR-N for disialylated core 2 *O*-GalNAc structures (Figures 2 and S1).

### Interaction of SLBR-N with 3′SLn: A Ligand- and Protein-Based Study

**Figure 3:**
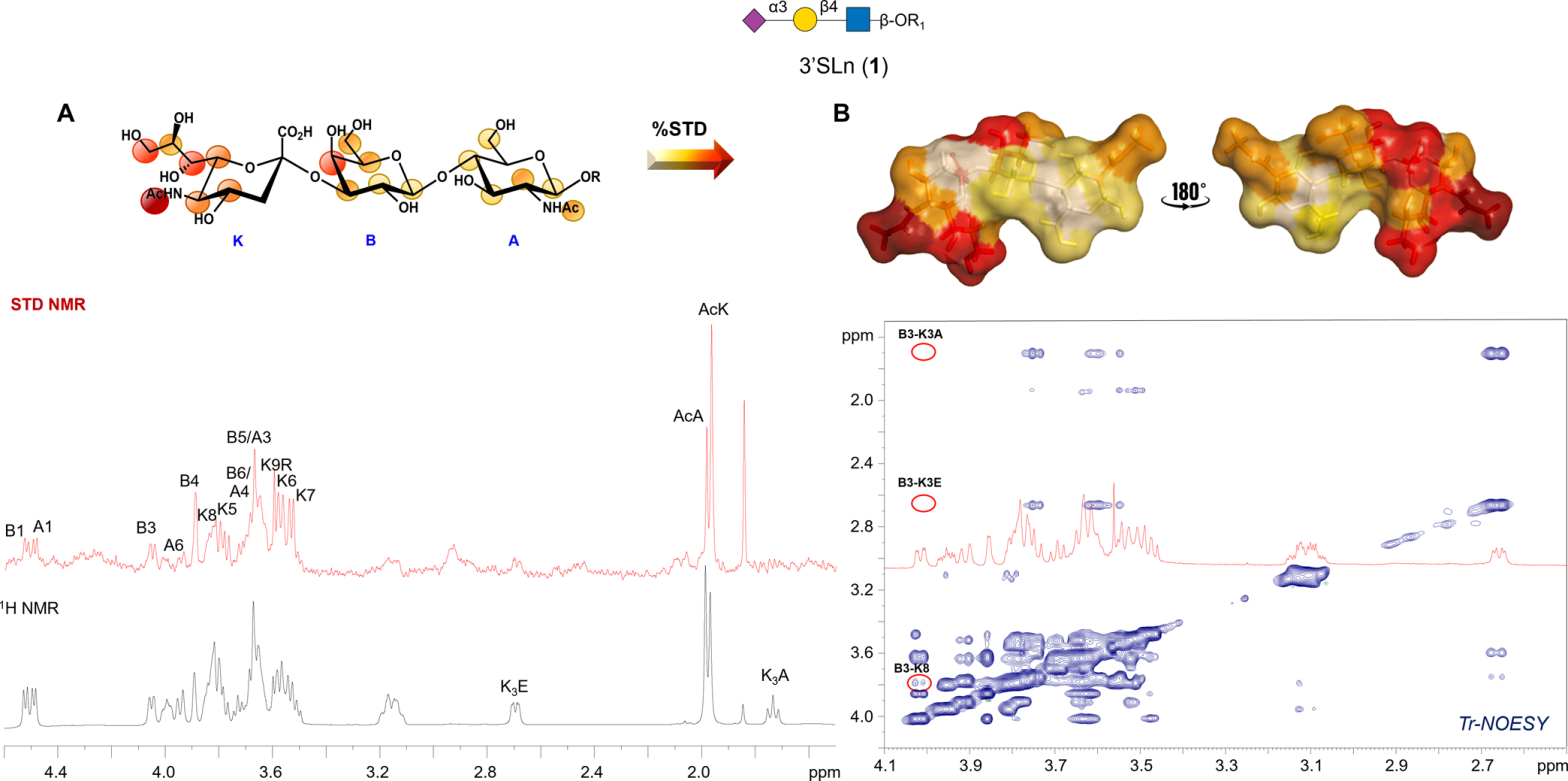
NMR results from the 3′SLn ligand perspective in interaction with SLBR-N. (A) STD NMR analysis of SLBR-N and ligand **1** with an indication of ligand epitope mapping calculated by (*I*_0_ – *I*_sat_)/*I*_0_, where (*I*_0_ – *I*_sat_) was the signal intensity in the STD-NMR spectrum (red) and *I*_0_ was the peak intensity of the off-resonance spectrum (black). The highest STD signal referring to the Ac group of Neu5Ac was set to 100%, and the other protons were normalized to this value. (B) Bioactive conformation of ligand **1** as obtained by the tr-NOESY spectrum; the ligand surface was colored according to the STD effects.

The molecular recognition features of 3′SLn (ligand **1**) by SLBR-N were unveiled by a combination of NMR and MD simulations. First, saturation transfer difference (STD) NMR confirmed the protein–ligand binding and mapped the recognized epitope, identifying the ligand protons in closest proximity to SLBR-N (Figure 3A). The acetyl group of sialic acid (AcK) received the highest magnetization from the receptor and was set to 100%; the relative STD intensities of the other protons were calculated accordingly. STD NMR experiments identified an extended binding epitope, involving all of the sugar moieties, with protons belonging to sialic acid and galactose units giving the strongest STD response, diagnostic of their closer proximity to the receptor (Figure 3A). The conformational behavior of **1** in both free and bound states was explored by NOESY and tr-NOESY experiments. The conformational flexibility for α2–3 sialoglycans depends on the behavior of the torsion angles around the Neu5Ac-α-(2–3)-Gal glycosidic linkage, namely, φ (C1–C2–O–C3′) and ψ (C2–O–C3′–H3′). Therefore, an almost stable ψ angle (around −11°), and a φ torsion oscillating around −60°, 180°, and 60°, were diagnostic of an equilibrium between the −g, t, and g conformers, respectively;^25,26^ this equilibrium between different conformational states was indeed confirmed by NOESY analysis on **1** in the free state (Figure S2 and Table S1). The bioactive conformation adopted by ligand **1** upon binding was then explored by tr-NOESY experiments. A detailed analysis of key trNOE contacts indicated a propensity of **1** for adopting the −g conformation upon binding to SLBR-N (Figure 3B and Table S1). This was supported by the key NOE between H3 Gal and H8 Neu5Ac (B3–K8) and the absence of NOEs between H3 Gal and the diastereotopic H3 protons of Sia, indicative of the 3′SLn propensity to adopt in the bound state a φ torsion at the Neu5Ac-Gal glycosidic linkage around −60°. Therefore, NMR binding experiments confirmed that **1** displayed an extended recognized epitope and underwent conformer selection upon binding to SLBR-N.

Protein-based NMR experiments were used to evaluate the interaction from the protein viewpoint. Triple-labeled ^2^H^13^C^15^N SLBR-N was expressed in *E. coli*, and then the protein resonances were assigned by 3D NMR experiments (Figure S3 and Supporting Information). Despite the low molecular weight (∼23 kDa), deuteration and the acquisition of spectra with the TROSY-scheme were required to extend the *T*_2_ relaxation times, due to the observed aggregation propensity of the protein at the concentration used for the NMR experiments. The evaluation of the binding of ligand **1** to SLBR-N was performed by titrating the ligand to the solution of the [U-15N] protein (Figure S4A) and following the variation in chemical shift and/or signal intensity of the cross-peaks in the 2D ^1^H–^15^N TROSY-HSQC spectrum of the protein.

Interestingly, the presence of **1** induced both a decrease in cross-peaks intensity (Figure S4B) and a chemical shift perturbation (CSP, Figure S4C) of some signals of SLBR-N at substoichiometric concentrations of the ligand. These variations allowed one to describe the binding region of SLBR-N in the presence of **1** (Figure S4D). In particular, the signals affected by the largest decreases in intensity were assigned to amino acids located on the CD loop (V284 and E285) and mainly to residues of the F strand (I335–R338) (see Scheme S1 and Figure S4A,B). Of note, signals corresponding to residues V284, E285, Y336, and T337 almost disappeared in the presence of low concentrations of the ligand (50 μM).

This result agreed with the expected binding mode of ligand **1**, because Y336 and T337 belonged to the φTRY consensus motif of SLBR-N and were bound to the sialic acid, while V284–E285 are on the adjacent CD loop. At the same time, the CSP analysis (Figure S4C) showed that residues of the F strand, and, in particular, the arginine and tyrosine in the φTRY consensus motif (R338 and Y339), as well as the adjacent residues of the EF loop (V332 and V333), experienced a large CSP. Changes in the chemical shifts of residues Y255, G260, L366, and N283, the latter belonging to the CD loop as well as M352 and E362 of the FG loop, were also observed. Of note, I335 and R338 were influenced by both CSP and variation of cross peak intensity. Furthermore, D254, Y255, and L366 of the β-strands close to the F-strand and M352 and E362 of the FG loop were indirectly affected by the interaction occurring between the neighboring amino acids and **1**, thus varying their chemical environment.

**Figure 4:**
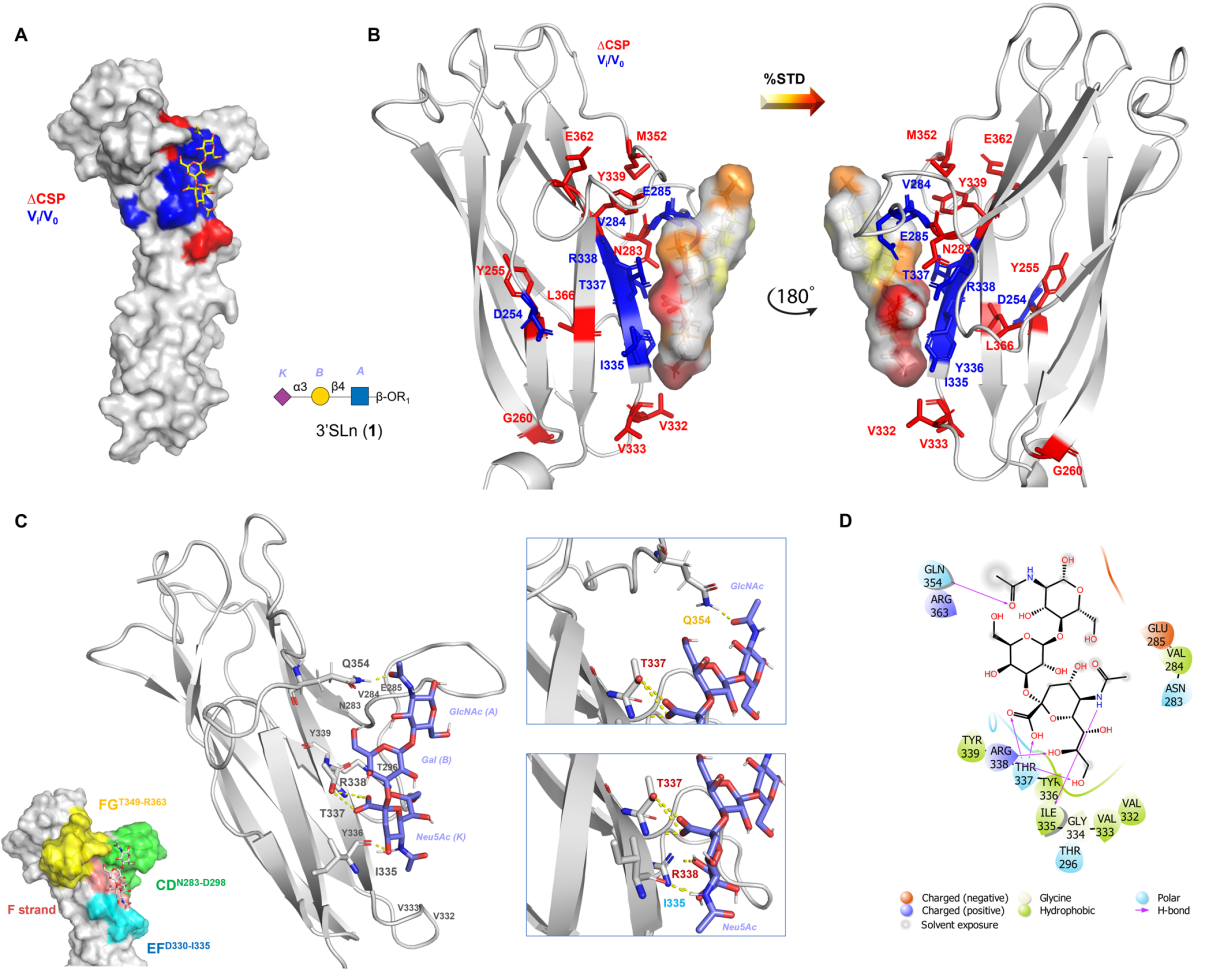
3D model of the SLBR-N–**1** complex. (A) 3D complex with protein surface colored according to chemical shifts perturbation (in red) and intensity decreases (in blue) detected by protein-based NMR titration. (B) 3D views of the SLBR-N–**1** complex: the amino acids involved in the interaction as revealed by protein-based NMR experiments were represented as sticks; the ligand surface was colored according to the STD edit code. (C) Representation of the SLBR-N–**1** complex with the CD, EF, and FG loops and F strand highlighted in green, cyan, yellow, and pink, respectively. Different views highlighting the H-bonds monitored by MD simulation were shown (in the zoom, the amino acids were colored according to the loops and F strand legend). (D) 2D plot of interactions occurring at the SLBR-N–**1** interface.

Overall, the spectral changes experienced by SLBR-N upon the addition of ligand **1** were in agreement with a protein–ligand binding affinity in the low micromolar range (see also native MS and ITC analyses in Figures 2B and S1). The combination of ligand- and protein-based NMR experiments allowed one to model **1** into the binding site of SLBR-N, and MD simulation analysis of the complex was performed to monitor the complex stability and dynamics of the binding in solution (Figures 4 and S5). Representative MD poses were then subjected to CORCEMA-ST (complete relaxation and conformational exchange matrix analysis of saturation transfer) analysis,^27^ a program that calculates the predicted STD-NMR intensities for proposed molecular models of a ligand–receptor complex; the calculated and measured STD values can be then compared and used to validate a given complex. Notably, the calculated STDs, predicted by CORCEMA-ST analysis, matched well with those experimentally observed, therefore confirming the reliability of the 3D model (Figures 4 and S5D).

Therefore, the SLBR-N–3′SLn 3D model showed a full accommodation of the ligand into the receptor binding pocket defined by the Siglec domain, with the sialic acid residue exhibiting the most stable and significant interactions.

### Interaction of SLBR-N with sTa-Thr

ESI–MS (Figure 2) and NMR experiments (Figure S6) revealed that sialyl-T-antigen (sTa-Thr, ligand **2**) was poorly recognized by SLBR-N. From a ligand perspective, the epitope mapping showed ligand **2** recognition by SLBR-N with the sialic acid residue mostly involved in the interaction (Figure S6A) with respect to the other residues, as was further confirmed by the contacts observed along the MD simulation (Figure S7). The CSP and the reduction of signal intensity were detected during the NMR titration of [U–^15^N] SLBR-N with ligand **2** (Figure S6B–D); most of the intensity decreases and chemical shift variations were found in the Siglec domain region, and some residues of the Unique domain experienced a slight CSP. In particular, the residues on the F strand (I335, Y336, T337, and R338), containing the φTRY consensus motif, experienced a decrease in signal intensity, and all, but T337, which disappeared completely, experienced chemical shift changes. However, these effects were observed at only a high molar excess of the ligand, meaning a weak interaction between SLBR-N and ligand **2**.

These results were in accordance not only with the data derived by the MS analysis (Figure 2) but also were corroborated by computational studies. The weak affinity of ligand **2** for SLBR-N detected by ESI–MS experiments was indeed proven by the root-mean-square deviation (RMSD) observed along the trajectory of the MD simulation (Figure S7). Although ligand **2** remained in the SLBR-N binding pocket, the RMSD plot indicated a variation of the ligand coordinates along the MD trajectory as compared to the starting frame (Figure S7B); indeed, the absence of significant interactions between SLBR-N and **2** allowed a dynamic behavior of the ligand within the Siglec domain, differently from ligand **1** (Figures 4 and S5). As an example, the carboxylate group of Neu5Ac made H-bonds with T337 and could also establish a salt bridge with R338. Overall, the F strand of SLBR-N containing a φTRY consensus motif, together with I335, was the main portion of the protein in contact with ligand **2**, particularly with the sialic acid, while the rest of the ligand established fewer contacts with the receptor. However, the global interactions at the SLBR-N–ligand **2** interface were not significatively reproduced along the trajectory, suggesting the formation of a weak affinity complex (Figure S7).

### Preference for 3′SLn versus sTa-Thr: Competition NMR Experiments and Monosialylated Core 2 *O*-Glycans

**Figure 5:**
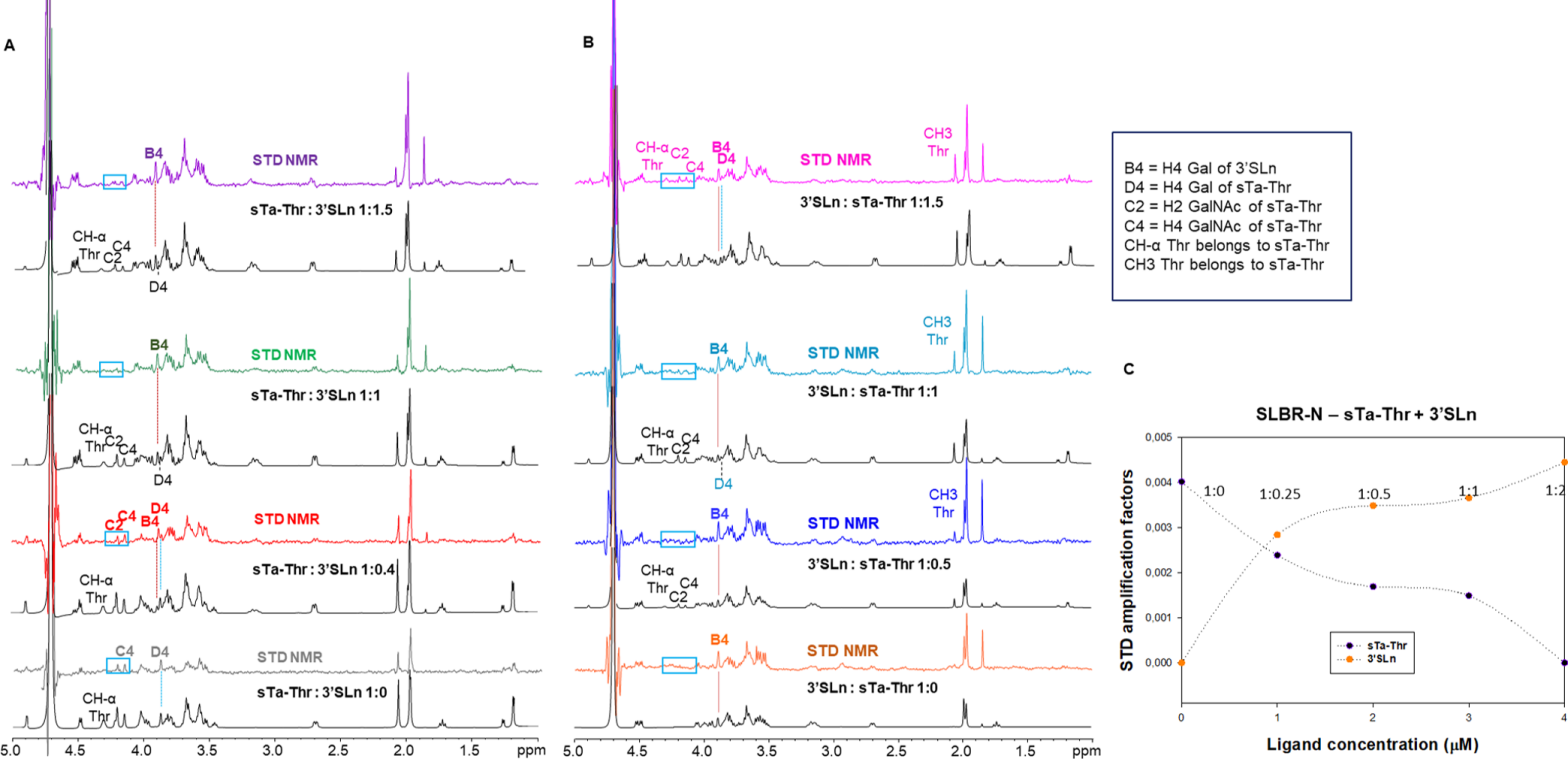
STD-derived competition experiments to compare the molecular binding of SLBR-N with ligands **1** and **2**. Two competition experiments were considered: the addition of ligand **1** to a mixture of SLBR-N + ligand **2**, and the addition of ligand **2** to a mixture of SLBR-N + ligand **1**. (A) STD NMR and off resonance spectra of the mixture containing SLBR-N (20 μM) and ligand **2** (2 mM) titrated with increasing concentrations of ligand **1** (from 800 μM to 3 mM). Key protons of the ligands were indicated in the STD NMR spectrum. At equal molar ligands ratio (1:1), the STD signals of ligand **2** disappeared, favoring the protein binding to ligand **1**. (B) STD NMR and off resonance spectra of the mixture containing SLBR-N (20 μM) and ligand **1** (2 mM) that was titrated with increasing concentrations of ligand **2** (from 500 μM to 3 mM). Key protons of the ligands were indicated in the STD NMR spectrum. STD NMR signals of ligand **2** were absent until an equal molar ligand ratio; low signals from threonine were detected only at a high molar excess of ligand **2**, likely deriving from nonspecific interactions. (C) Graphic view of the titration of SLBR-N–ligand **2** with ligand **1**: the STD amplification factors of H4 Gal belonging to ligand **2** (purple) and to ligand **1** (orange) were calculated at different ligands ratios (*x* axis), clearly confirming the preference of SLBR-N for **1** with respect to **2**.

The higher affinity of SLBR-N for 3′SLn versus sTa-Thr (ligands **1** and **2**) measured by native ESI–MS and ITC experiments was also evaluated and confirmed by STD NMR competition experiments (Figure 5). The mixture of SLBR-N and ligand **2** was titrated with ligand **1** (Figure 5A). The addition of the competing high-affinity ligand was determined by an increase of STD signals of ligand **1** and a progressive reduction, until complete disappearance, of STD signals of ligand **2** (Figures 5A and S8). On the other hand, when the mixture of SLBR-N and ligand **1** was titrated with ligand **2** (Figures 5B and S9), almost no STD NMR response from sTa protons was observed. These data clearly indicated that ligand **2** was weakly recognized by SLBR-N, which in turn exhibited high affinity for **1**. Competition experiments were also performed by protein-based NMR experiments, confirming the above ligand-based NMR results. Indeed, upon addition of ligand **1** to the SLBR-N–ligand **2** mixture (Figure S10), CSP and a large decrease in cross peak intensity were detected, and the signals affected were almost the same observed during SLBR-N titration with ligand **1** (see above and Figure 4), further proof of a higher affinity complex. Finally, by comparing the root-mean-square fluctuation (RMSF) of the CD, EF, and FG loops of free and bound SLBR-N, a major stabilization was observed for the complex with ligand **1** (Figure S11).

Binding studies with monosialylated core 2 *O*-glycans (Scheme S1 and Figure 2, ligands **3** and **4**), alternatively terminating with 3′SLn and sTa-branch, further confirmed the preference of the protein for the sialyl-lactosamine epitope. These data revealed that both monosialyl core 2 *O*-glycans could be recognized by SLBR-N, with Sia’s accommodating in the binding pocket according to the corresponding trisaccharides ligands **1** and **2** (see details in the Supporting Information and in Figure S12).

### Interaction of SLBR-N with Disialylated Core 2 *O*-Glycans

**Figure 6:**
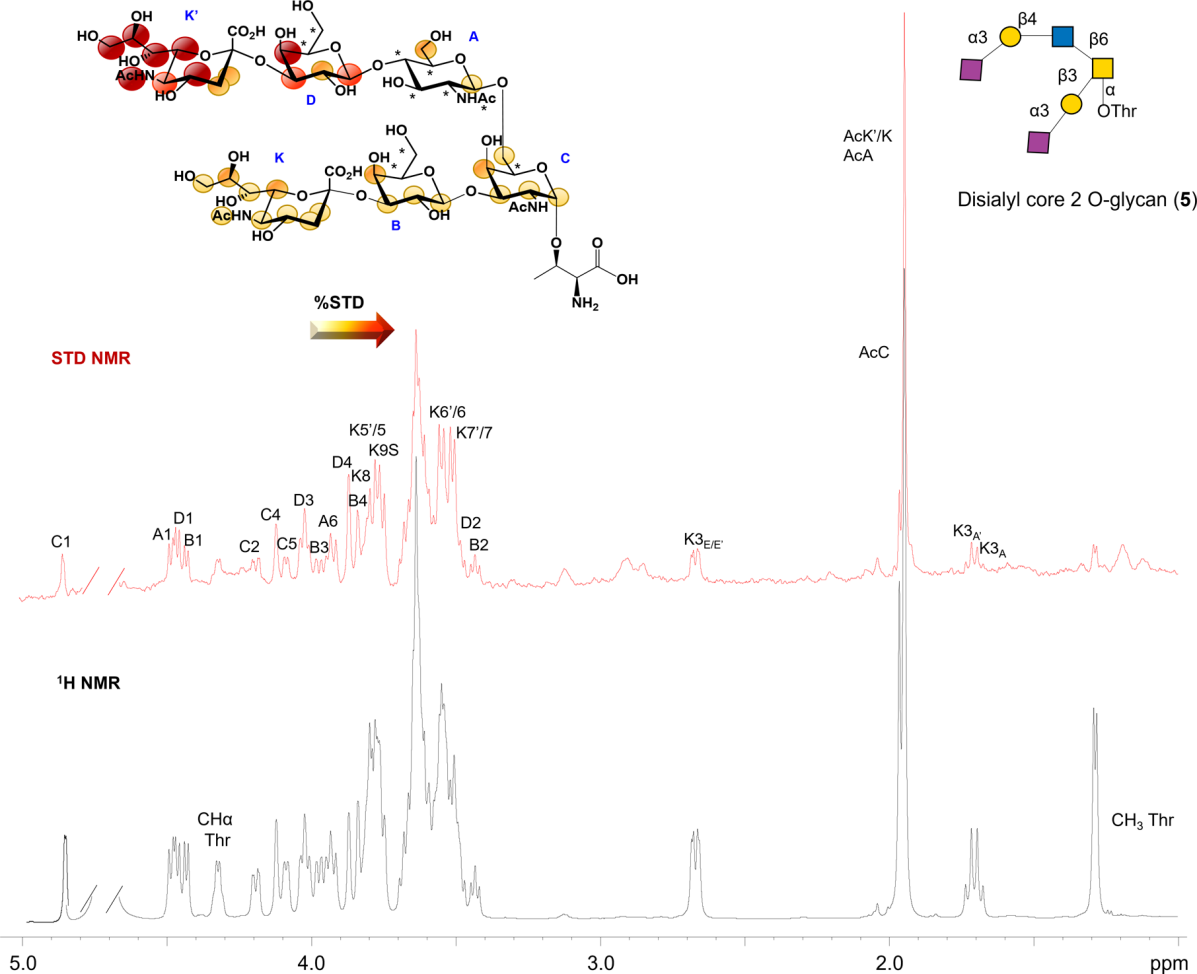
STD NMR analysis of SLBR-N and ligand **5**. The epitope mapping was calculated by (*I*_0_ – *I*_sat_)/*I*_0_, where (*I*_0_ – *I*_sat_) was the signal intensity in the STD-NMR spectrum (red) and *I*_0_ was the peak intensity of the off-resonance spectrum (black). Sialic acid residues were not distinguishable by NMR, and their epitope mapping was derived according to protein NMR experiment theoretical STD effects calculated by CORCEMA-ST. The overlapped protons were indicated with asterisks on the chemical structure.

ESI–MS, ITC, NMR, and computational studies provided important information on how disialylated core 2 *O*-glycans (ligand **5**) could be recognized by SLBR-N. STD NMR experiments explored the binding and mapped the interacting epitope, showing that ligand **5** was extensively involved in the binding to SLBR-N (Figure 6).

**Figure 7:**
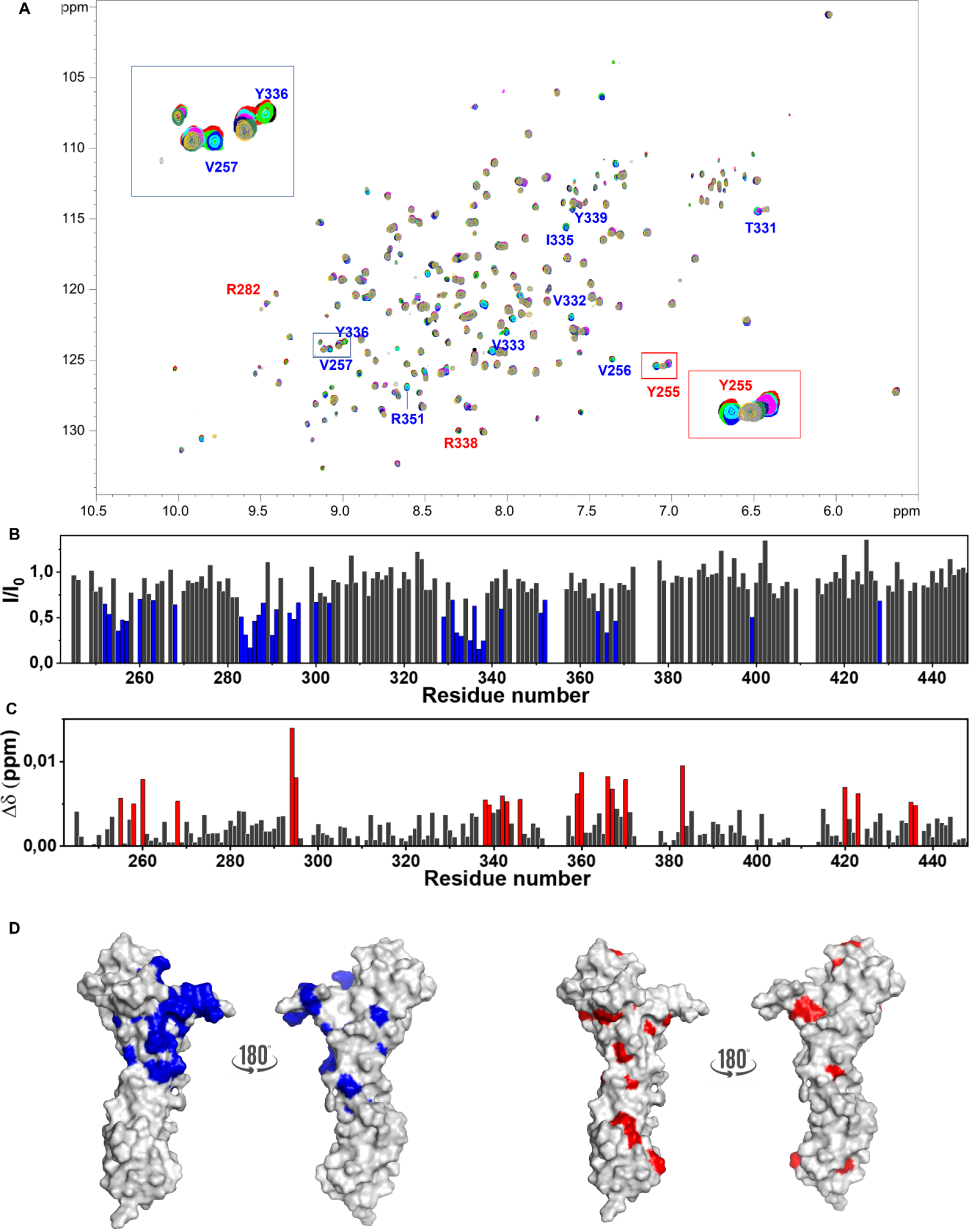
Protein-based NMR analysis of the SLBR-N–ligand **5** mixture. (A) 2D ^1^H ^15^N TROSY-HSQC NMR spectra of the SLBR-N free (black) and in the presence of 12.5 μM (red), 25 μM (green), 50 μM (blue), 100 μM (cyan), 200 μM (magenta), 400 μM (dark blue), 800 μM (dark green), 1600 μM (yellow), and 2080 μM (gray) of ligand **5**. The addition of the ligand leads to CSPs (red labels) and the decrease of intensity (blue labels) of some amino acids. Solutions were prepared in phosphate buffer pH 7.4 acquired on a spectrometer operating at 1.2 GHz at 298 K. (B) Plot representing the decreases in signal intensity per residue of SLBR-N in the presence of 200 μM of ligand **5** with respect to the protein (200 μM). The residues experiencing the largest signal intensity decrease (Y255, V256, V257, E268, N283, V284, E285, L286, D287, K288, T290, N291, Y294, L295, T296, L300, S303, L329, V332, V333, I335, Y336, T337, R338, A342, R351, M352, F364, L366, V368, V399, and Y428) have been highlighted in blue. (C) Plot of the CSP of the protein in the presence of 200 μM disialylated core 2-O glycan with respect to protein experiments that explored the binding and mapped the interacting epitope (Y258, E268, Y294, L295, R338, Y339, A342, T343, A346, D359, G360, L366, T367, S370, T383, Q420, T423, T435, and I436), which have been highlighted in red. (D) Surface representations of a model of the protein with highlighted residues experiencing the largest decreases in signal intensity (in blue) and the largest CSP (in red) in the presence of 100 μM ligand **5**.

Furthermore, titration of [U–^15^N] SLBR-N with ligand **5** (Figure 7) showed that the area of the protein affected upon binding was more extensive if compared to ligands **1** and **2** (Figures S4 and S6). Indeed, ITC results showed that the binding was enthalpically driven, mainly due to the numerous establishments of hydrogen bonds between SLBR-N and ligand **5** (Figure S1B). The enthalpy measured by SLBR-N–ligand **5** binding was more favorable than that obtained by SLBR-N–ligand **1** interaction (−82.8 ± 1.65 vs −25.5 ± 0.979 kJ/mol); this result was reasonable given the higher number of contacts established at the interface with the protein, driven by the longer glycan chain of **5**, with two Sia residues.

Amino acids defining the binding site experienced a decrease in intensity as well as CSPs and mainly belonged to the Siglec domain (Figure 7).

**Figure 8:**
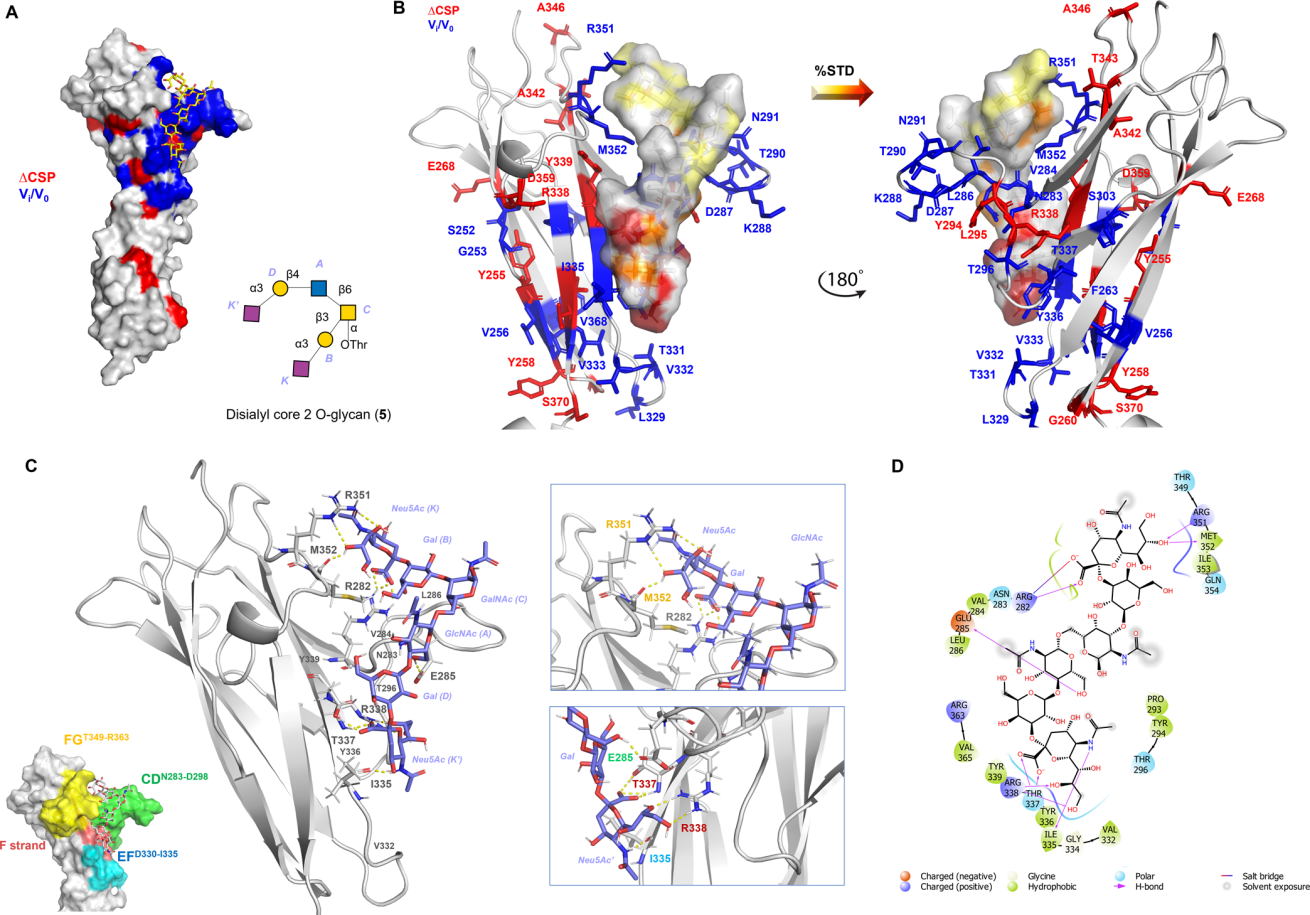
3D model of the SLBR-N–**5** complex. (A) 3D complex with protein surface colored according to chemical shifts perturbation (in red) and intensity decreases (in blue) detected by protein-based NMR titration. (B) 3D views of the SLBR-N–**5** complex: the amino acids involved in the interaction as revealed by protein-based NMR experiments were represented as sticks; the ligand surface was colored according to the STD edit code. (C) Representation of the SLBR-N–**5** complex with CD, EF, and FG loops and the F strand highlighted in green, cyan, yellow, and pink, respectively. Different views highlighting the H-bonds monitored by MD simulation were shown (in the zoom, the amino acids were colored according to the loops and F strand legend). (D) 2D plot of interactions occurring at the SLBR-N–**5** interface.

In particular, residues Y336 and R338 of the φTRY consensus motif, as well the proximal residues, were affected by both decreases in signal intensity and CSP. On the basis of NMR data, MD simulation, and CORCEMA-ST (Figures S13 and S14), a 3D model of the complex was obtained. The entire ligand **5** was accommodated into the protein, with the 3′SLn branch bound to the F-strand of the Siglec domain, the region containing the φTRY sequence of SLBR-N (Figure 8).

In detail, Y336, T337, and R338 established H-bond interactions with Neu5Ac of the 3′SLn branch, including carboxylate and acetyl groups as well as the OH at positions 8 and 9 of the glycerol chain. On the other hand, Neu5Ac of the sTa branch established H-bonds with R351 and M352, both affected by an intensity decrease during protein titration, and a salt bridge with R282, affected by CSP. The above results indicated that the ligand was entirely splayed on the protein surface, with the 3′SLn arm sitting in the (main) protein binding site and the sTa branch establishing further polar interactions with amino acids of CD and FG loops. A recurrent H-bond between E285 and OH at position 6 of GlcNAc was further detected by MD simulation. Interestingly, this amino acid residue was affected by a decrease of signal intensity by NMR titration and significantly stabilized the protein–ligand interaction.

Indeed, as for ligand **6**, sulfation of position 6 of GlcNAc drastically reduced the affinity of the ligand to SLBR-N, as reported by ESI–MS data (Figure 2A). NMR binding data indicated that, although both arms of the ligand were recognized, the 3′SLn branch was the main engaged by the protein upon binding (Figure S15). By comparing the NMR and MD data of sulfated (ligand **6**) and nonsulfated (ligand **5**) *O*-glycans (Figure S16), it was possible to observe a similar accommodation into the binding site of SLBR-N, although more populated poses from MD cluster analysis and more stable contacts were found in the complex with ligand **5**, making it the preferred ligand (see also Discussion).

## Discussion

We here focused on the molecular details of host *O*-glycan recognition by the streptococcal Siglec-like adhesin SLBR-N of *S. gordonii* strain UB10712. Such protein-glycan systems are dynamic, intricate, and challenging complexes driven by a combination of forces including hydrogen bonds, van der Waals, electrostatic, and hydrophobic interactions, which were here investigated by combining complementary approaches and techniques. The combination of flow cytometry, native ESI–MS, ITC, NMR spectroscopy, and MD simulations allowed one to detect ligand binding affinities and to identify branched disialylated core 2 *O*-glycan as the preferred substrate of SLBR-N. Native mass spectrometry has emerged as an important tool in studying the structure and properties of the complexes. It is a versatile method used to evaluate noncovalently driven assemblies in a native-like folded state, providing information on the relative binding affinities and stoichiometry. Here, we could quantify weak interactions by using minimal amounts of protein and ligands, revealing the entire distribution of ligand-bound states and providing the *K*_D_ between SLBR-N and ligands **1**–**6**. The investigation, without the need for labeling or cross-linking, was performed on picomoles of material, while at the same time offering high resolution and a speed of analysis in the time scale of milliseconds. The order of magnitude of the association constants provided by the native mass spectrometry analysis is consistent with the NMR and ITC data. The addition of submillimolar/millimolar concentrations of the ligands to the investigated protein resulted in a sizable decrease in the intensity of the signals from the residues forming the specific binding site. Also, in the presence of a large excess of the ligands, new signals corresponding to the protein–ligand complexes were not observed.

Moreover, small chemical shift variations are observed for some signals. This behavior is consistent with an affinity in the micromolar range. Affinity constants calculated by ESI–MS were also in accordance with ITC results, confirming the preference of SLBR-N for ligand **1** with respect to **2** and showing ligand **5** as the preferred epitope. The NH backbone of the protein was assigned under per-deuteration conditions of expression and by performing 3D triple-resonance NMR experiments; the assignment was essential for the analysis of ligand interactions. In all complexes here studied, the main binding site was always comprised of the region containing Y^336^T^337^R^338^Y^339^ amino acids, a consensus motif common to all Siglec-like adhesins. We further revealed that SLBR-N was the only adhesin of its family that contained an isoleucine (I335) in the EF loop that formed a recurrent key contact with the acetyl group of sialic acid, confirming the importance of the amino acids on the F strand in the binding with sialoglycans (see Scheme S1). The main binding site of SLBR-N exhibited a clear preference for accommodating sialyl-lactosamine portion, as unveiled by native MS and ITC, showing the strongest affinities for the ligands containing the 3′SLn branch with respect to the sTa branch, and also confirmed by competition NMR experiments (Figures 2, 5, and S8–S10).

Interestingly, in complex core 2 *O*-glycans, both 3′SLn and sTa arms contributed to the interaction, as evidenced (i) by a wider binding region of SLBR-N when titrated with this longer ligand, (ii) by the STD NMR effects, variously and extensively involving most, if not all ligand protons of core 2 glycan (Figure 6), and (iii) by the numerous contacts monitored by MD, including polar, electrostatic, and hydrophobic interactions, which involved both sialic acids at the termini of the ligand arms (Figure 8). This is consistent with the thermodynamic parameters obtained by ITC, and in particular with the high negative enthalpy (Δ*H* −82.8 kcal/mol, Figures 2 and S1B) that confirmed the proposed 3D model (see also CORCEMA data, Figure S14) that involves additional contacts with respect to the shorter ligand **1**. Despite the large variation of enthalpies, the Δ*G* values were similar, because this latter term was compensated by a negative entropy. In carbohydrate recognition events, an entropic penalty is typically observed, due to glycan loss of flexibility from the free state to the interaction with a protein. Overall, for **1** and **5**, the reduction of entropy was compensated by a favorable enthalpic contribution. This was observed for ligand **1** that experienced a conformational selection upon binding around the glycosidic linkage (Figure 3B and Table S1). Furthermore, ligand **5** undergoes a stronger loss of conformational entropy upon complex formation, and this reduction of flexibility (Figures S1B and S14) supports the unfavorable entropy.^28^ Thus, in complex core 2 *O*-glycans, accommodation of the 3′SLn branch into the main binding site represents the driving event that allows the interaction of the sTa branch to SLBR-N. Interestingly, while the carboxylate group of Sia belonging to the 3′SLn branch formed an H-bond with T337 of the main binding site, the carboxylate group of the Sia belonging to the sTa branch established a salt bridge with R282, a crucial interaction observed in sialoglycans recognition by mammalian Siglec’s.^29,30^ Moreover, the presence of the sulfate group on the 3′SLn moiety of the core glycan did not influence the general orientation of the ligand into the SLBR-N binding site; rather, it precluded the binding of the GlcNAc residue to E285, due to electrostatic repulsions (Figure S16). Indeed, previous studies demonstrated that E295R substitution generated preference for the sulfated ligand with respect to the OH at position 6 of GlcNAc.^19^ In the 3D models provided in this study, the negatively charged sulfate was instead found in contact with K288 of the CD loop of SLBR-N, but this interaction was weakly repeated along the MD simulation and, for this reason, considered negligible.

**Figure 9:**
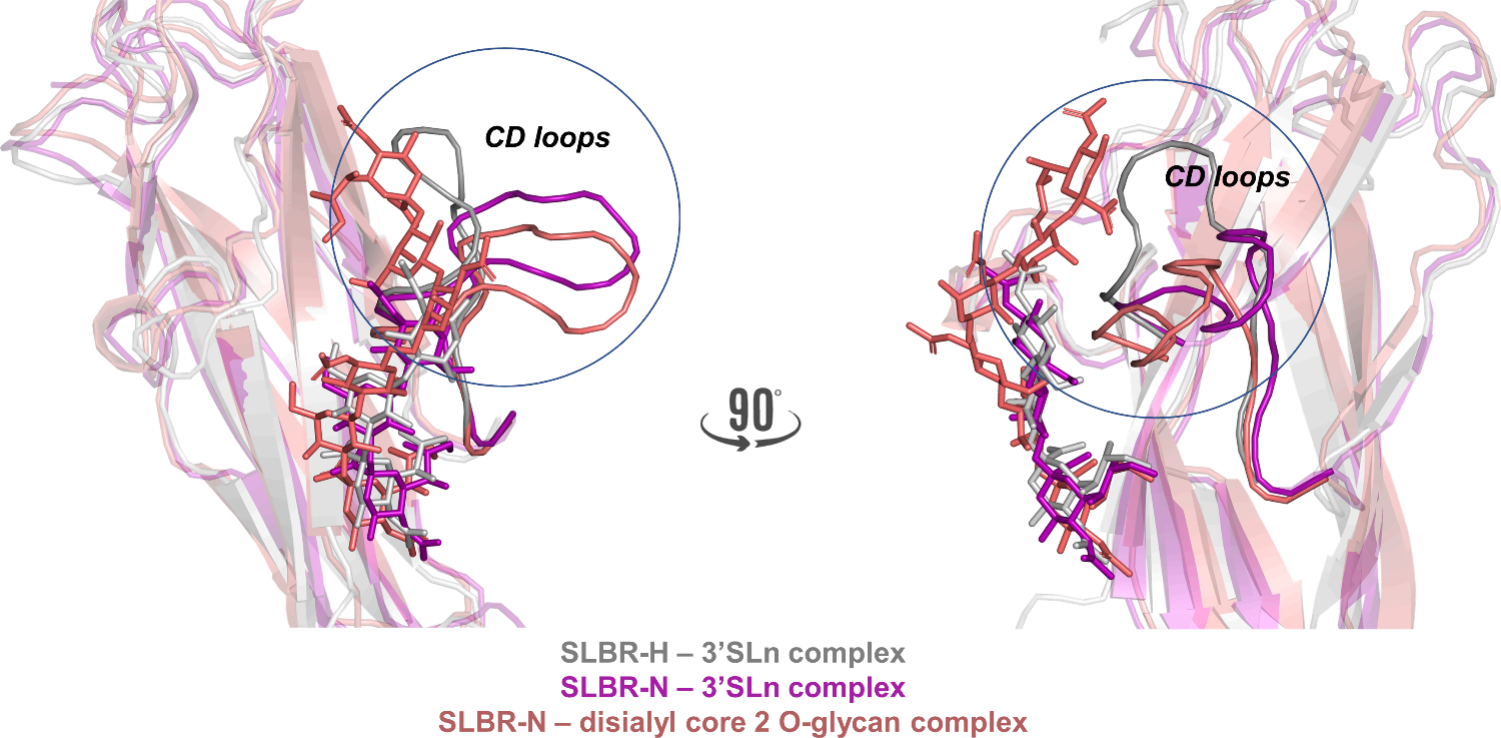
Comparison of the SLBR-H and SLBR-N binding modes with ligands **1** and **5**. The more open CD loop conformation of SLBR-N accommodates the complex disialyl core 2 *O*-glycan, which would cause steric hindrance with SLBR-H.

Despite the high sequence identity (80%) between SLBR-N and SLBR-H, Siglec-like adhesins from *S. gordonii* UB10712 and Challis, respectively, the ligand binding preferences are different, and their selectivity depends on the loop composition and flexibility.^14,20^ Indeed, differently from SLBR-H,^15^ the CD loop of SLBR-N assumed a more open conformation as shown by our 3D models of protein–ligand complexes (Figure 9) that defined a wider binding pocket with both 3′SLn and sTa branches and, consequently, accommodated the entire complex core 2 *O*-glycans. These data likely explain why the binding epitope of SLBR-H corresponds to a monosialylated core 1 *O*-glycan, supporting the results shown by Narimatsu et al.^18^ The strongest preference of SLBR-N for the disialylated ligand was instead ascribable to the extended interactions with the amino acids on the F strand (YTRY) and those on CD, EF, and FG loops detected by NMR and MD. Among these, the hydrogen bonds between I335^EF^ and the acetyl group of Sia of the 3′SLn branch, E285^CD^ with OH6 of GlcNAc, R351^FG^ and M352^FG^ with the glycerol chain of Sia belonging to the sTa branch, as well as the hydrophobic interactions that involved V284^CD^, L285^CD^, V332^EF^, and M352^FG^ all contribute to an increased affinity of SLBR-N for the disialyl core 2 *O*-glycan.

Based on the above results, we can assess that the structural features of the binding between SLBR-N and host *O*-glycans are now clear at the atomic level. Our findings allowed us to determine the ligands epitope maps and their bioactive conformations in the receptor binding site as well as to define the amino acids of the protein binding pocket. Collectively, these outcomes described the architecture and the nature of the molecular interactions, providing the 3D models of different sialoglycans in complex with SLBR-N (Figure S17). Structural biology studies are always a necessary preliminary step toward the drug design and the chemical synthesis of entities that can tune a biochemical/immunological process. The versatility of the combined techniques used in this work, which include native mass spectrometry, calorimetry, biophysical, NMR, and computational approaches, represents a fundamental step to develop a site-specific interaction-based design strategy. By our approach encompassing an array of biophysical techniques, we have given new insights for developing potential high affinity mimetics that can maintain oral commensalism while hindering systemic streptococcal infections.

## Supporting Information Available

Additional figures and experimental procedures employed for the production and assignment of SLBR-N, interaction with sialoglycans, MS, ITC, NMR ligand conformation experiments, and CORCEMA-ST (PDF)
Figure S17 (PDF)
Figures S1–S16 (PDF)

## Abbreviations

SRRP: serine-rich repeat glycoproteins
BR: binding region
IE: infective endocarditis
CSC: cancer stem cell
HIV: human immunodeficiency virus
ESI–MS: electrospray mass spectrometry
ITC: isothermal titration calorimetry
NMR: nuclear magnetic resonance
MD: molecular dynamic
CORCEMA-ST: complete relaxation and conformational exchange matrix analysis of saturation transfer
HSQC: heteronuclear single quantum coherence
TROSY: transverse relaxation optimized spectroscopy
NOESY: nuclear Overhauser effect spectroscopy
ROESY: rotating frame Overhauser enhancement spectroscopy
CSP: chemical shift perturbation
RMSD: root-mean-square deviation
RMSF: root-mean-square fluctuation
3′SLn: 3′-sialyl-*N*-acetyllactosamine
sTa: sialyl-T-antigen
sLeC: sialyl Lewis C
sLeX: sialyl Lewis X
