# Supplementary material for: Molecular Insights into O-Linked Sialoglycans Recognition by the Siglec-Like SLBR-N (SLBRUB10712) of Streptococcus gordonii
Source: ACS Cent Sci. 2024 Feb 7;10(2):447–59. doi: 10.1021/acscentsci.3c01598 (PMC10906241; doi:10.1021/acscentsci.3c01598)
Supplement: Supplementary file 1 — oc3c01598_si_001.pdf [file oc3c01598_si_001.pdf]

Supporting information

Molecular insights into O-linked sialoglycans recognition by the Siglec-like SLBR-N ($SLBR_{UB10712}$) of *Streptococcus gordonii*

Cristina Di Carluccio[1], Linda Cerofolini[2], Miguel Moreira[1], Frédéric Rosu[3], Luis Padilla-Cortés[2], Giulia Roxana Gheorghita[2,4], Zhuojia Xu[5], Abhishek Santra[6], Hai Yu[6], Shinji Yokoyama[7], Taylor E. Gray[9], Chris D. St. Laurent[9], Yoshiyuki Manabe[7], Xi Chen[6], Koichi Fukase[7], Matthew S. Macauley[9,10,] Antonio Molinaro[1,7], Tiehai Li[5], Barbara A. Bensing[8], Roberta Marchetti[1*], Valérie Gabelica[3], Marco Fragai[2], and Alba Silipo[1*]

1. Department of Chemical Sciences, University of Naples Federico II, Italy
2. Magnetic Resonance Centre (CERM), CIRMMP and Department of Chemistry "Ugo Schiff", University of Florence, Sesto Fiorentino, Italy
3. IECB Institut Européen de Chimie et Biologie, France
4. Giotto Biotech s.r.l., Sesto Fiorentino, Italy
5. Shanghai Institute of Materia Medica, Chinese Academy of Sciences, China
6. Department of Chemistry, University of California-Davis, USA
7. Graduate School of Science, Osaka University, Japan
8. San Francisco Veterans Affairs Medical Center and University of California, San Francisco, USA
9. Department of Chemistry, University of Alberta, Edmonton, Alberta, Canada
10. Department Medical Microbiology and Immunology, University of Alberta, Edmonton, Alberta, Canada

*email roberta.marchetti@unina.it; silipo@unina.it

Table of Contents

Isothermal titration calorimetry	S3
Interaction of SLBR-N with 3'SLn	S3
SLBR-N NMR assignment and titration experiments	S3
lecular interaction of SLBR-N and monosialyl core2 O-glycans	S4
Scheme 1	S5
Scheme 2	S6
Table S1	S7
Experimental section	S8-S11
Supporting figures	S12-S13
Supporting references	S14

Isothermal titration calorimetry

Isothermal titration calorimetry (ITC) experiments allowed to measure the heat released by the interaction between SLBR-N and ligands **1**, **2**, **5** (Figure S1B). The quantitative analysis has been performed by titrating a fixed concentration of SLBR-N with increasing amounts of ligand of interest at constant temperature and pressure. Through ITC titration, affinity constants were determined together with thermodynamic terms of enthalpy (ΔH), entropy (ΔS)and Gibbs energy (ΔG)of the binding.

In the case of ligand **2**, it was not possible to obtain an ITC curve, because the interaction with the protein was too weak to be reliably detected (data not shown).[1] In the case of ligands **1** and **5**, the interactions with SLBR-N were enthalpically driven and the affinity constant values were determined, resulting in accordance with K_D obtained by ESI-MS (Figure S1). In both cases, the formation of the complexes was favorable, with negative enthalpic contribution, due to the occurrence of non-covalent interactions, as hydrogen bonds, when SLBR-N bound each ligand. However, the enthalpy measured by SLBR-N-ligand **5** binding was more favorable than that obtained by SLBR-N-ligand **1** interaction (-82.8 ± 1.65 vs -25.5 ± 0.979 kJ/mol); this result was reasonable given the higher number of contacts established at the interface with the protein driven by the presence of two sialic acids in ligand **5**. Despite the large variation of enthalpies, ΔG values were similar, because this latter term was compensated by a negative entropy. In carbohydrate recognition events, an entropic penalty is typically observed, due to glycan loss of flexibility from the free state to the interaction with a protein.[2] A stronger loss of conformational entropy was obtained upon the formation of complex SLBR-N-ligand 5 ($-T\Delta S$ = 56.2 kJ/mol) with respect to ligand **1** ($-T\Delta S$ = 3.21 kJ/mol). Overall, for ligands **1** and **5**, the binding processes were spontaneous ($\Delta G < 0$), with a reduction of entropy that was compensated by a favorable enthalpic contribution. Furthermore, affinity constants calculated by ITC confirmed the stronger binding of SLBR-N for ligand **5**, as highlighted by ESI-MS.

Interaction of SLBR-N with 3'SLn

As previously reported,[3] ligand **1** can populate three different conformational minima in the free state with Neu5Ac-Gal glycosidic torsion angle φ (C1-C2-O-C3') at -60°/180°/60°. In the presence of SLBR-N, ligand **1** underwent a conformer selection with φ of -60°, as shown by NMR experiments and monitored by MD simulation of the complex in explicit water (Figures 3B, S2 and Table S1). Ensemble average interproton distances were extracted and translated into NOE contacts according to a full-matrix relaxation approach; the absence of $H3_{eq}$/ $H3_{ax}$ Neu5Ac – H3 Gal NOEs and, instead, the presence of H8 Neu5Ac – H3 Gal NOE were indicative of a φ of -60° 3'SLn in the bound state (Figure 3B, Table S1). The average distances obtained for the MD simulation from $\langle r^{-6} \rangle$ values were compared to those collected experimentally, and notably an excellent accordance between the experimental and calculated data was found.

SLBR-N NMR assignment and titration experiments

Acquisition of NMR spectra for backbone assignment of SLBR-N was first attempted on the [U-^{13}C,^{15}N] isotopically enriched protein; nevertheless, SLBR-N aggregation shortened T_2 relaxation times and induced signals broadening, thus impairing NMR studies. Therefore, the protein was expressed as triple-labeled ([U-^{2}H^{13}C^{15}N] SLBR-N), since deuteration considerably extended the T_2 relaxation times, resulting in narrow signals in the NMR spectra. Firstly,^{1}H-^{15}N TROSY (Transverse Relaxation-Optimized Spectroscopy) HSQC NMR experiments were recorded on ^{15}N-labelled SLBR-N to obtain the NH fingerprint. The 87% of the protein residues were then assigned from the analysis of triple resonance experiments (Figure S3).

Once assigned, the protein binding site was assessed upon titration of SLBR-N with glycans of interest. Several ^{1}H^{15}N TROSY-HSQC spectra were recorded: the reference spectrum was acquired on the free

protein; then, increasing amounts of the ligand were added to the mixture and TROSY-HSQC spectra were acquired accordingly. The ligands affected the chemical environment of the surrounded amino acids, therefore highlighting their involvement in the binding event. An example of chemical shift variation for SLBR-N when interacting with ligand **1** was shown in Figure S4.

Molecular interaction of SLBR-N and monosialyl core2 O-glycans

STD NMR experiments were performed to determine the molecular binding between SLBR-N and monosialylated ligands corresponding to the different branches of the disialyl core2 O-glycan (ligands **3** and **4**, data not shown). In both cases, the STD recognition profile indicated that the ligand **3** was preferentially recognized by the protein with respect to ligand **4**. Both ligands were then modeled into the binding site of SLBR-N, with the sialic acid interacting with the F strand containing YTRY consensus sequence (Figure S12A-B). According to the STD NMR data, a major stability of the loops, especially for the CD and EF loop, was observed in the complex formed by SLBR-N and ligand **3** (Figure S12C). Overall, although both monosialylated glycans could bind to SLBR-N, the recognition profile for the ligand **3** was more defined with respect to **4**, meaning a preference of SLBR-N in binding the ligand containing 3'SLn branch.

Scheme 1. The general organization of SRR adhesins and the amino acid sequences of SLBR-B, SLBR-H and SLBR-N (Siglec and Unique domains) of *S. gordonii*. SP is the signal peptide; SRR1 and SRR2 are the serine-rich regions 1 and 2; BR stands for ligand binding region and CWA is the cell wall anchor domain. The φTRY consensus motif is evidenced by the red square.

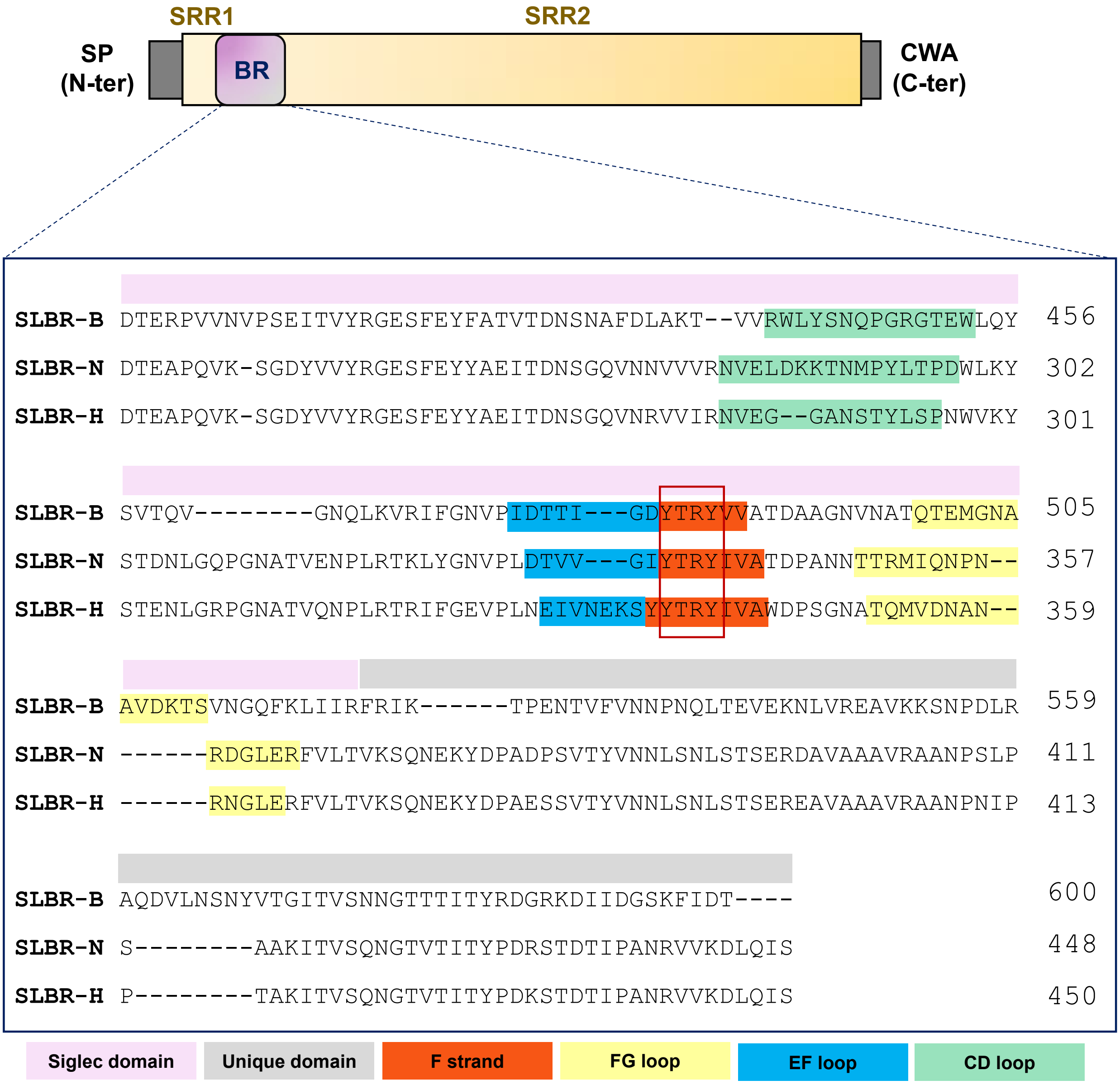

Scheme 2. Ligands studied in interaction with SLBR-N. R_1 is ethanolamine, R_2 is threonine, R_3 is methoxybenzene.

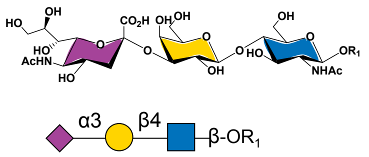

3'SLn
(ligand 1)

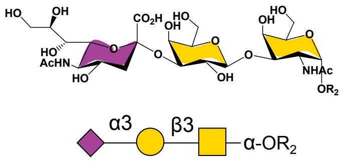

sTa-Thr
(ligand 2)

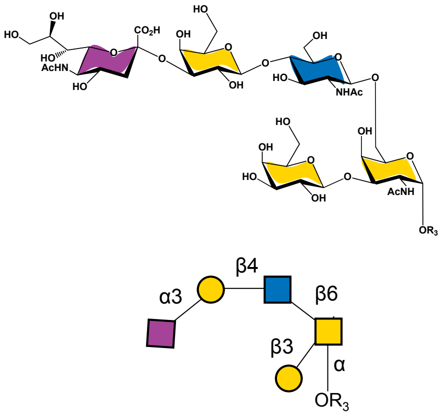

Monosialyl core 2 O-glycan
(ligand 3)

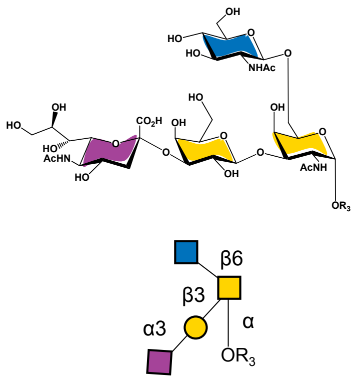

Monosialyl core 2 O-glycan
(ligand 4)

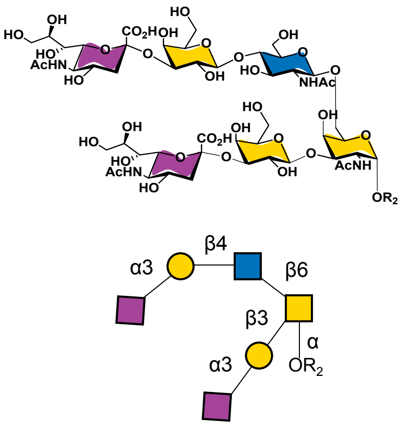

Disialyl core 2 O-glycan
(ligand 5)

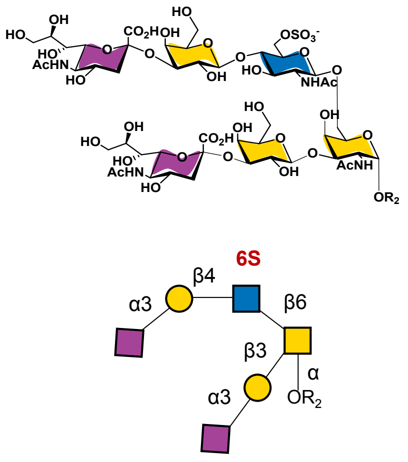

Sulphated Disialyl core 2 O-glycan
(ligand 6)

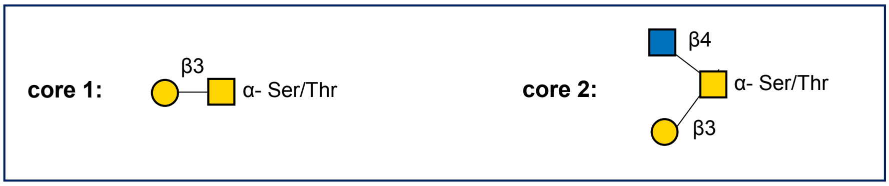

Table S1. Key ^{1}H-^{1}H distances to determine ligand **1** bioactive conformation. The values in the table were obtained by tr-NOESY experiment at 298K, using H1-H5 of GlcNAc as reference. In bold the preferred conformation of ligand **1** bound to SLBR-N (ϕ = -60°).

^{1}H-^{1}H distance	Theor. Φ -60°	Theor. Φ 60°	Theor. Φ 180°	Exp. free NOESY	Exp. bound tr-NOESY
$H3_{ax}$ Neu5Ac – H3 Gal	**4.10**	3.11	2.18	2.86	/
$H3_{eq}$ Neu5Ac – H3 Gal	**4.40**	2.03	3.40	3.65	/
H8 Neu5Ac – H3 Gal	**3.36**	6.17	4.31	3.41	3.10
$H3_{ax}$ Neu5Ac – H4 Gal	**5.40**	2.13	4.45	/	/
$H3_{eq}$ Neu5Ac – H4 Gal	**4.15**	2.25	5.15	/	/

Experimental section

Ligands synthesis.
Ligands **1-6** were synthesized as previously reported.[4,5]

Flow citometry.
Creation of the Sialyltransferase Knockout U937 cells. Custom crRNA (Integrated DNA Technologies; IDT) was designed to first target human ST6GAL1 (sequence = CAGATGGGTCCCATACAATT). U937 cells were seeded at 500,000 cells per well the day of transfection in a 6-well tissue culture plate, in 1.3 mL growth media (RPMI containing 10% FBS and 100 U/mL penicillin-streptomycin). For one well of a 6-well plate, 20 pmol of Cas9 nuclease (IDT), 20 pMol of ATTO-550 labeled crRNA:tracrRNA duplex (IDT), 8uL Cas9 Plus reagent, and 16 µL CRISPRMAX reagent (Thermo Fisher) in 665 µL Opti-MEM medium (Thermo Fisher), were incubated together for 15 minutes, and then added to the cells. One day after transfection, cells were removed from the plate, washed, resuspended in 300 µl of cell sorting medium (HBSS, 2% FBS, 1 mM EDTA), and stored on ice until sorting. Cells were sorted on a FACSMelody cell sorter (BD Biosciences). The brightest 8% of cells stained with ATTO-550 were sorted into 96-well plates containing growth media at one cell per well. Cells were grown for 14 days until colonies were large enough to be screened. These were screened by flow cytometry using fluorescein-conjugated Sambucus Nigra Lectin (SNA, 1:750, Vector Laboratories) to identify ST6GAL1 KO clones. Genomic DNA was isolated from potential KO clones by combining 100,000 cells with 96 µL DirectPCR (cell) lysis reagent and 4 µL Proteinase K (Viagen Biotech). This was incubated for 2 hours at 55°C, followed by 30 minutes at 85°C. An ~700bp sequence of ST6GAL1 surrounding the CAS9 cut site was amplified by PCR using 1 µL of cell lysis, FW primer GGCCTCAGGCTGTACCTTG, and RV primer GATCTGCGCCTTCTGCTTAG. ST6GAL1 KO clones were then confirmed by identifying CRISPR generated INDELs adjacent to the CAS9 cut site by Sanger sequencing of the PCR products. crRNA targeting ST3GAL1 (sequence= GCCCTTCAAGACCATCGACT) was used on one ST6GAL1 KO clone identified above, using the same methodology. Potential ST6/ST3GAL1 double knockouts were identified by screening with peanut agglutinin (PNA, 1:2000), and confirmed using Sanger sequencing (Fw primer CACATGGGCTGACCCTTTCTAGTGG, RV primer TGGCTAGACTCCCTTGAGGTCATGA). crRNA targeting ST3GAL4 was then used (sequence= AATACCACACCATGACGACC) on one ST6/ST3GAL1 double KO clone identified above, using the same methodology. Potential ST6GAL1/ST3GAL1/ST3GAL4 triple knockouts were identified by screening with "Siglec-like binding region N" (SLBR-N, 2µg/mL)), and confirmed using Sanger sequencing (Fw primer ACCAGACCAAGAGACGCCTTGGATG, RV primer CCCTCATGGAAACACTCCGTACCAGC).
SLBR-N Transformation: A plasmid encoding glutathione S-transferase- (GST) tagged SLBR-N was expressed in Escherchia coli (E. coli) BL21 DE3 competent cells. BL21 DE3 cells were thawed on ice and GST-SLBR-N (2 ng) was added to the cells without mixing. After incubation on ice for 30 min, the mixture was heat shocked at 42 °C for exactly 30 seconds and then placed on ice for 5 minutes. SOC Outgrowth media (950 µL) was added to the freshly transformed E. coli cells and placed in a shaking incubator at 37°C for 1 hour. On a warm agar plate containing 50 ng/mL ampicillin, 50 µL cell mixture was spread and incubated overnight at 37°C.
Protein Expression: A single colony was inoculated into 6 mL LB media containing 50 ng/mL ampicillin and incubated with shaking overnight at 37°C. On the following day, all 6 mL of the growing culture was added to 500 mL LB media with 50ng/mL ampicillin and incubated with shaking until the optical density reached approximately 0.6 (~3-4 hours). Protein expression was then induced with the addition of isopropylthio-β-D-galactoside (IPTG, 1 mM) and incubated overnight at 25 °C. On the following day the culture was centrifuged at 6000 x g for 15 min. The supernatant was discarded, and the pellet was resuspended in 50 mL 1X phosphate buffered saline (PBS), centrifuged at 6000 x g for 15 min and the supernatant was discarded.
Protein purification: Cell pellets were resuspended in 40 mL lysis buffer (50 mM Tris HCl, pH 8, 25 mM NaCl, 2 mM EDTA, 0.1% Triton-X 100) and combined with lysozyme (300 µg/mL) from chicken egg white. Mixture was shaken at 37°C for 1 hour and lysed by vigorously sonicating until a homogenous solution was achieved. The mix was then centrifuged for 30 min at 12,000 x g and the supernatant was passed through a 0.22 µm filter. A GSTrap FF 1mL high performance column (GE healthcare) was equilibrated with 5 column volumes (CV) of wash buffer (1X PBS, 10mM DTT, pH 7.4) at a flow rate of 1 mL/min. The lysate was then applied to the column in its entirety. The column was washed with 10 CV wash buffer and GST-SLBR-N was eluted with 6 mL elution buffer (20mM phosphate buffer, 150mM NaCl, 10mM reduced glutathione, 10mM DTT, pH 8.0) in 0.5 mL fractions. Fractions containing protein were identified with nanodrop and dialyzed into 1X PBS. SLBR-N was quantified with Bradford assay.

Alexafluor-647 labelling: SLBR-N (200 µg) was combined with 950 µL PBS and 50 µL 1M sodium bicarbonate (pH 8) and 16.6 µg Alexafluor-647 NHS ester. The solution was left to react for 1 hr at room temperature in the dark, and subsequently dialyzed into PBS.

Native mass spectrometry.

The protein was dissolved in water from Biosolve (UPLC-MS grade). For desalting, Amicon ultracel-10 centrifugal filters (Merck Millipore Ltd) with 10-kDa molecular weight cut-off were used. Several passes with 100 mM NH_4OAc followed by pure water to reach satisfactory desalting were performed. Protein solution was prepared at 50 µM in 50 mM NH_4OAc. Dilution to 5 µM was performed before injection.

Experiments were performed on an Agilent 6560 DTIMS-Q-TOF instrument (Agilent Technologies, Santa Clara, CA), with the dual-ESI source operated in the negative ion mode. A syringe pump flow rate of 190 µL/h was used. Capacitance diaphragm gauges are connected to the funnel vacuum chamber and to the drift tube. An in-house modification to the pumping system allows better equilibration of the pressures: a Edwards E2M40 vacuum pump (Edwards, UK) is connected to the source region with two Edwards SP16K diaphragm valves connected to the front pumping lines, while an Edwards nXR40i vacuum pump is connected to the Q-TOF region. The helium pressure in the drift tube was 3.89 ± 0.01 Torr, and the pressure in the trapping funnel was 3.79 ± 0.01 Torr. The pressure differential between the drift tube and the trapping funnel ensures only helium is present in the drift tube. The acquisition software version was B.09.00. All spectra were recorded using soft source conditions. The tuning parameters of the instrument (electrospray source, trapping region and post-IMS region (QTOF region)) are optimized as described elsewhere.[6] The source temperature was set at 220 °C and the source fragment or voltage was set to 320 V. The trapping time was 1000 µs and release time 200 µs. Trap entrance grid delta was set to 2 V.

Isothermal titration calorimetry.

SLBR-N was dialyzed against PBS H2O pH 7.4 and ligands were prepared in the dialysate in order to avoid buffer mismatch between the solutions. All measurements were performed with a MicroCal PEAQ-ITC (Malvern Instrument) at temperature of 15°C. Samples were prepared using 80 µM of SLBR-N (without GST tag) and concentrations of stock solutions were calculated based on dry weights of ligands. The protein was loaded into the cell, and, for each measurement, ligand was added to the protein solution through a micro syringe under stirrer speed of 750 rpm. The reference power was set at 10 µW and the analysis was recorded with low feedback. The time duration between the 19 injections of ligand was at 150 seconds and 2 µl of ligand titrant was added for each injection. Binding isotherm of the calorimetric titration was obtained, with the enthalpy of the interaction plotted against the protein/ligand molar ratio. Data were fitted in a model of *One set of sites* with stoichiometry n = 1. Thermodynamic parameters were then calculated by the following equations[7]:

$$\Delta G = RT \ln K_D$$
$$\Delta G = \Delta H - T\Delta S$$

Protein expression and purification.

The Siglec and Unique domains of SLBR-N were expressed as fusion protein with a GST-tag. A plasmid based on a pGEX vector and containing the coding sequence of this fusion protein transformed in *E. coli* BL21 (DE3) cells was used to express the fusion protein. Luria broth (LB) media supplemented with 50 ug/mL of ampicilin was inoculated with the plasmid-containing *E. coli* cells and the cell culture was allowed to grow at 37ºC under agitation (180 rpm). When the cell cultured reached the exponential phase (A_{600nm} ~ 0.6) the heterologous protein expression was induced with 1mM of isopropyl-1-thio-d-galactopyranoside (IPTG) and allowed to occur overnight at 18 ºC. Subsequently the cells were harvested by centrifugation at 7500 rpm for 20 min at 4ºC and resuspended in phosphate buffer saline (PBS) pH 7.4 supplemented with 1mM DTT and ABSF protease inhibitors. The soluble protein was purified using a Glutathione Sepharose 4B column (GE Healthcare), eluted with 10 mM GSH and 10 mM DTT in 50 mM Tris-HCl, pH 8.0 (elution buffer). To further purify the resulting protein in PBS pH 7.4, a final size-exclusion chromatography was used by using a HiLoad 16/60 Superdex 75 pg column (GE healthcare). The unlabeled fusion protein GST-SLBR-N was used for ligand-based NMR purposes.

The expression of double-labeled [$^{13}C^{15}N$] and triple-labeled [$^{2}H^{13}C^{15}N$] SLBR-N followed the same approach with the following modifications. For double-labeled SLBR-N the cells were grown in 2L of M9 minimal media supplemented with 2.8g $^{15}NH_4Cl$ and 6 g of ^{13}C-enriched glucose. Triple-labeled SLBR-N was expressed using Silantes OD2 solution rich growth media instead.

Labeled GST-SLBR-N proteins were purified by following the same protocol used for non-labeled GST-SLBR-N. Briefly, after cell harvest and resuspension, the cells were lysed by sonication and the lysate was cleared by centrifugation at 17000 rpm for 40 min at 4ºC. The lysate was loaded on a glutathione affinity chromatography column (Glutathione Sepharose 4B column, GE Healthcare) and the protein was eluted with 10 mM GSH and 10 mM DTT in 50 mM Tris-HCl, pH 8.0. Subsequently, for labeled GST-SLBR-N proteins, a further desalting step against PBS pH 7.4 was performed by using a High Trap Desalting 26/10 column to remove the reducing agents present in the elution buffer coming from the GST-trap column. To remove the GST-tag, the labeled GST-SLBR-N proteins were incubated with 1ug/uL of Factor Xa protease for 16h at RT. A second GST-affinity chromatography was employed to remove the cleaved GST-tag from the protein preparation. Finally, a size-exclusion chromatography was used to further purify the resulting protein using a HiLoad 16/60 Superdex 75 pg column (GE healthcare) previously equilibrated with PBS pH 7.4.

Backbone resonance assignment of SLBR-N.

NMR protein experiments for backbone assignment were acquired on a sample of [U- 2H13C15N] SLBR-N at the concentration of 340 μM in 100 μL of water buffered solution (20 mM sodium phosphate, pH 7.5, 0.02% NaN_3, 10% D2O, and 0.1 mg/mL of protease inhibitors) in a 3 mm Shigemi tube. A series of triple resonance experiments with a TROSY scheme tr-HNCA, tr-HNCACB, tr-HN(CO)CA and tr-HN(CO)CACB were recorded at 298 K for the protein assignment. The NMR spectra were acquired on Bruker's Avance™ NEO 700, 900 and 1200 MHz spectrometers, equipped with triple resonance TCI cryo-probes. Data acquisition and processing were performed with TOPSPIN 4.1.1 software and spectra were analyzed by using CARA program.[8]

Protein-based NMR titration of SLBR-N with different ligand.

For ligands binding studies, 2D $^{1}H^{15}N$ TROSY-HSQC NMR experiments[9] were recorded on samples of [U-15N] SLBR-N in 200 μL at the concentration of 200 μM in water buffered solution (20 mM sodium phosphate, pH 7.5, 0.02% NaN_3 and 10% D2O) using Bruker's Avance™ NEO 1200 MHz spectrometer, equipped with triple resonance TCI and TXO cryo-probes. The spectra were acquired using 32 scans with 2048 data points in t2, 128 increments in the indirect dimension (t_1), a recycle delay of 1.2 sec, and the temperature was kept at 298 K. The interaction of SLBR-N protein with the ligands has been investigated by adding increasing amounts of each ligand to the solution of the protein to reach the ligand concentrations in solution of: 12.5, 25, 100, 200, 400, 800 and 1600 μM. The 2D 1H- 15N TROSY-HSQC spectra were acquired after the addition of each ligand aliquot. Data acquisition and processing were performed with TOPSPIN 4.1.1 software and CARA program. Chemical shift perturbations (CSPs) were evaluated with the formula $\Delta\delta = \frac{1}{2}\sqrt{\Delta\delta_H{}^2 + (\Delta\delta_N/5)^2}$.[10,11]

Ligand-based NMR experiments.

Saturation Transfer Difference (STD) and transferred-NOESY NMR experiments were recorded on a Bruker AVANCE NEO 600-MHz equipped with a cryo-probe and data acquisition and processing were performed with TOPSPIN 4.1.1 software. Samples were prepared in phosphate saline deuterated buffer (10 mM Na_2HPO_4, 2.7 mM KCl, 137 mM NaCl, 10 mM NaN_3, pH 7.4) at 298 K. [D4](trimethylsilyl)propionic acid, sodium salt (TSP, 1 %) was used as internal reference. GST-SLBR-N/ligands molar ratios varied from 1:50 to 1:90.

STD NMR experiments were acquired with shaped pulse train for saturation on f2 channel alternating between on and off resonance with 20 ms spinlock pulse applied to suppress protein signals. The acquisition was set with 65 k data points and 112 number of scans. The protein resonances were selectively irradiated by 40 Gauss pulses with a length of 50 ms, using the off-resonance pulse frequency at 40 ppm and on-resonance pulses at 7.5 ppm and 0 ppm. The STD NMR spectra were acquired at saturation time of 2 s. STD NMR effects were calculated by the ratio $(I_0 - I_{sat})/I_0$, where I_{sat} is the relative intensity of the STD NMR signal and I_0 the peak intensity of an unsaturated reference spectrum (off-resonance). The highest STD NMR response was set to 100% while all the other STD signals were normalized to this value to obtain the ligands' epitope maps.[12]

2D ^{1}H-^{1}H NOESY experiments were carried out by using data sets of 2048x512 points and 200 ms as mixing time. Proton – proton cross relaxation rates were calculated by integration of cross peaks normalized against the corresponding diagonal peak. ^{1}H-^{1}H distances were calculated using the following equation: $r_{ij} = r_{ref}\sqrt[6]{\sigma_{ref}/\sigma_{ij}}$, where r_{ij} is the unknown distance to be estimated, r_{ref} is the reference interproton distance, σ_{ref} is the cross-relaxation rate of the NOE cross peak of interest and σ_{ij} is the cross-relaxation rate of reference.[13]

Competition measurements by STD NMR experiments.

STD competition NMR experiments were performed to establish the SLBR-N preference toward ligands 1 and 2.[14,15] Spectra were acquired on a Bruker AVANCE NEO 600-MHz equipped with a cryo-probe. Data acquisition and processing were performed with TOPSPIN 4.1.1 software. Samples were prepared in in phosphate saline deuterated buffer (10 mM Na_2HPO_4, 2.7 mM KCl, 137 mM NaCl, 10 mM NaN_3, pH 7.4) at 298 K, using 0.05 mM trimethylsilylpropanoic acid (TSP) as an internal reference. Two competition experiments were considered: the addition of ligand 1 to a mixture of GST-SLBR-N with ligand 2, and the addition of ligand 2 to a mixture of GST-SLBR-N with ligand 1. In all experiments, the concentration of GST-SLBR-N was fixed at 20 μM (dilution factors were adjusted at each addition of ligand) and the protein:ligand ratio was 1:100.

Competition measurements by protein-based NMR experiments.

Competition experiments were performed by adding ligand **1** to a mixture of [U-^{15}N] SLBR-N and ligand **2**. Increasing aliquots of ligand **1** (to reach the ligand concentration of: 40, 80, 160, 320, 640, 1280, 2560 μM) were added to the solution of SLBR-N (160 μM) in the presence of an excess of ligand **2** (1280 μM). 2D ^{1}H-^{15}N TROSY-HSQC spectra were recorded at 298 K after each addition on a Bruker AVANCE III HD NMR spectrometer, operating at 950 MHz (^{1}H Larmor frequency) and equipped with a triple resonance cryo-probe.

MD simulations

Glycans were generated on GLYCAM website.[16] The non-standard sTa linked to threonine residue was parametrized. 100 ns and 500 ns MD simulations were performed by using AMBER 18.[17] The prmtop and inpcrd files were generated with the tLEaP module. The force fields were GLYCAM06j-1 for carbohydrates parameters and protein.ff14SB for SLBR-N. No torsional restrictions were applied. Complexes were prepared properly before the MD simulation: counter ions were added to neutralize the systems by using the Leap module and octahedral boxes containing explicit TIP3P water molecules were considered. MD simulations were run by using the CUDA implementation of PMEMD in AMBER18.[18] Minimization steps of all complexes were performed using Sander. The smooth particle mesh Ewald method was used to depict the electrostatic attractions in the system while applying periodic boundary constraints and the grid spacing was 1 Å. The system underwent the first annealing gradually and gently over a 25-ps period from 100 °K to 300 °K. Throughout 50 ps, a steady temperature of 300 °K was maintained with progressively energy minimizations. The MD coordinates were gathered to acquire 1000 frames of the progression of the dynamics. Using the K-mean algorithm implemented in the ptraj module of the AMBER18 software, the trajectories were submitted to cluster analyses, in order to obtain the main representative poses. MD simulations were visualized by using VMD program.[19]

CORCEMA-ST analysis.

The complete relaxation and conformational exchange matrix analysis of saturation transfer (CORCEMA-ST) was used to compare theoretical and experimental STD data and to validate the 3D models of SLBR-N with ligands **1** and **5** by the calculation of R-NOE factors. The theory and the protocol of CORCEMA-ST was extensively described.[20] The pdb coordinates of the 3D models were the most representative poses obtained by MD simulations. The input parameters used for the CORCEMA-ST analysis were chosen according to the experimental conditions: i) saturation time of 2 s; ii) protein concentration of 20 μM; iii) ligand concentration of 2 mM; iv) dissociation constants according to ESI-MS/ITC experiments. The saturation in the aromatic region was assumed with the STD irradiation frequency at 7.5 ppm. In the case of ligand **5**, R-NOE of the 3D model was calculated excluding protons of sialic acid residues.

Supporting Figures legends

Figure S1. A) Native mass spectrometry analysis. Collisional cross section distribution of SLBR-N protein as a function of the charge state. Low charge states (12+, 13+, are compact (folded) and higher charge states become gradually more extended (unfolded). Soft conditions are used (fragmentor 320V, trap entrance grid delta 2V). C) MS determined equilibrium association constants (M^{-1}) for the different compounds and the SLBR-N, revealing the following ligand preference: : **5** >> **3** > **1/6** >> **4** > **2**. Protein concentration was 5 μM in 50 mM ammonium acetate and the starting ligand concentration was 20 μM. The equilibrium association constants were defined: K1=[1:1]/([protein]*[Lfree]) and K2=[2:1]/([1:1]*[Lfree]). The concentration of each species in solution at equilibrium was calculated using the ratio of intensities and the mass balance equations. The association constants of the interaction between SLBR-N and ligands **1-6** were listed in the table. B) Isothermal titration calorimetry analysis. Heat changes for titration of SLBR-N with ligands **1** (top) and **5** (bottom). Data fit to *One set of sites* interaction models and provided affinity constants and thermodynamic profiles of the interactions.

Figure S2. NOESY spectrum of ligand **1** in its free state at 298 K and mixing time of 600 ms.

Figure S3. 2D ^{1}H ^{15}N TROSY-HSQC NMR spectrum of the free SLBR-N in phosphate buffer pH 7.4 acquired on a spectrometer operating at 1.2 GHz at 298 K. The deuteration increased T_2 and allowed sharp and well resolved signals. Amino acid residues obtained by the protein assignment were indicated in the spectrum.

Figure S4. Protein-based NMR analysis of SLBR-N and ligand **1**. The addition of micromolar concentrations of 3'SLn to ^{15}N SLBR-N induced changes in intensity and CSPs of the amino acids interacting with the ligand, defining the binding site of the protein. A) 2D ^{1}H ^{15}N TROSY-HSQC NMR spectra of free 200 μM SLBR-N (black) and the protein in the presence of 100 μM 3'SLn (red). By the addition of ligand **1**, variations in chemical shifts of SLBR-N were observed. B) Plot representing the decrease in signal intensity per residue of SLBR-N in the presence of 100 μM of ligand **1** with respect to the free protein. C) Plot of the CSP of SLBR-N in the presence of 100 μM of ligand **1** with respect to the free protein. D) Surface representation of a model of the protein (PDB code: 6EFF) with highlighted in blue the residues experiencing the largest decreases in signal intensity (D254, Y255, V284, E285, I335, Y336, T337, R338) and in red the residues experiencing the largest CSP (Y255, G260, N283, V332, V333, I335, R338, Y339, M352, E362, L366) in the presence of 100 μM ligand **1**. The residues not assigned in the 2D ^{1}H ^{15}N TROSY-HSQC NMR of [^{2}H ^{13}C ^{15}N] SLBR-N were indicated in grey.

Figure S5. 3D model of SLBR-N – ligand **1** complex. A) φ and ψ dihedral angles around Neu5Ac-Gal and Gal-GlcNAc glycosidic linkages, respectively, indicating a conformer selection in accordance with tr-NOESY results. B) Root mean square deviation profiles of SLBR-N and ligand **1** evidencing the stability of the complex along MD simulation. C) Amino acids of SLBR-N affected by chemical shift variation experienced by NMR and those in contact with ligand by MD were represented as spheres. A good accordance between theoretical and experimental data was achieved. D) CORCEMA-ST analysis to compare theoretical and experimental STD effects of ligand **1**. R-NOE of 0.4 was obtained and the 3D model was then validated. Labels: K = Neu5Ac, A = GlcNAc, B = Gal.

Figure S6. Molecular recognition of ligand **2** by SLBR-N by NMR. A) STD NMR (red) and off-resonance (black) spectra and corresponding epitope mapping of ligand **2** recognized by SLBR-N; the presence of signals in the STD NMR spectrum was an indication of the binding. B) Plot representing the decrease in signal intensity per residue of SLBR-N in the presence of molar excess of ligand **2** (320 μM) with respect to the free protein (160 μM). The residues experiencing the largest decrease in signal intensity (Y255, V284, V332, I335, Y336, T337, R338, V365, V368, Q371, D397, R404, A406, T417, D434) have been highlighted in blue. C) Plot of the CSP of SLBR-N in the presence of 320 μM ligand **2**. The residues experiencing the largest CSP (Y255, V284, E285, V333, I335, Y336, R338, I353, S370, Q371, N372, A378, A398, T427, Y428, T433, I436) have been highlighted in red. D) Surface representation of a model of the protein (PDB code: 6EFF) with highlighted in blue the residues experiencing the largest decreases in signal intensity and in red the residues experiencing the largest CSP in the presence of ligand **2**.

Figure S7. MD analysis of SLBR-N and ligand **2**. A) 3D models of ligand **2** into the Siglec domain of SLBR-N, obtained by the cluster analysis. B) RMSD plot of SLBR-N and sTa-Thr: the ligand **2** moved into the protein binding site along the trajectory, showing a weak stability. C) 2D diagrams of the interaction between SLBR-N and ligand **2** of the main representations from the cluster analysis.

Figure S8. Ligand-based NMR competition of ligand **1** on the SLBR-N - ligand **2** mixture. A) ^{1}H NMR spectra acquired at increasing concentrations of ligand **1** to SLBR-N : ligand **2** (1 : 100). B) STD NMR spectra acquired at increasing concentrations of ligand **1** to SLBR-N : ligand **2** (1 : 100).

Figure S9. Ligand-based NMR competition of ligand **2** on the SLBR-N – ligand **1** mixture. ^{1}H NMR spectra acquired at increasing concentrations of ligand **2** to SLBR-N : ligand **1** (1 : 100).

Figure S10. Increasing aliquots of ligand **1**(to reach the ligand concentration of 40, 80, 160, 320, 640, 1280, 2560 μM, respectively) were added to the solution of SLBR-N (160 μM) in the presence of an excess of ligand **2** (1280 μM). A) Plot representing the decreases in signal intensity per residue of SLBR-N in the presence of 80 μM ligand **1** with respect to the protein (160 μM) in the presence of ligand **2** (1280 μM). The residues experiencing the largest signal intensity decrease (T245, Y255, I269, N272, V279, V284, E285, Y294, T296, V333, I335, R351, A398) have been highlighted in blue. B) Plot of the CSP of the protein in the presence of 80 μM ligand **1** with respect to protein (160 μM) in the presence of ligand **2** (1280 μM). The residues experiencing the largest CSP (T245, E246, Y258, G260, I269, V284, E285, K288, Y294, K301, G308, V332, V333, I335, R338, I353, N357, V365, L366, K369, S370, Q371, N372, A378, R396, D397, T427, Y428, T433, D434) have been highlighted in red. C) Surface representation of a model of the protein bound to ligand **1** with highlighted in blue the residues experiencing the largest decreases in signal intensity in the presence of 80 μM ligand **1** and 1280 μM ligand **2.** D) Surface representation of a model of the protein bound to ligand **1** with highlighted in red the residues experiencing the largest CSP in the presence of 80 μM ligand **1** and 1280 μM ligand **2.** E) CSP plots for V256 and T331 residues with respect to ligand **1** concentration obtained for the titration of free SBLR-N (empty circles) and for the titration of SLBR-N in the presence of 1280 μM ligand **2** (full circles). The CSP plot with respect to ligand **2** concentration (triangles) have been also included for comparison purpose. The presence of ligand **2** in solution decreased the affinity of ligand **1** for the protein, indicating competition between the two ligands for the same binding site.

Figure S11. RMSF profile of free SLBR-N (black), SLBR-N in complex with ligand **1** (green) and SLBR-N in complex with ligand **2** (red), with amino acids belonging to CD, EF and FG loops highlighted. A major stability of the loops, especially for the EF loop, was observed in the binding between SLBR-N and ligand **1**.

Figure S12. SLBR-N binding to monosialyl core2 O-glycans. A) 3D model of the interaction between SLBR-N and ligand 4. B) 3D model of the interaction between SLBR-N and ligand 3. C) RMSF profile of free SLBR-N (black), SLBR-N in complex with ligand **3** (green) and SLBR-N in complex with ligand **4** (red), with amino acids belonging to CD, EF and FG loops highlighted. The superimposition of the monosialylated ligands with the corresponding trisaccharides (ligands **1** and **2**) are also shown.

Figure S13. MD analysis of ligand **5** in the free state.

Figure S14. A) MD analysis of ligand **5** bound to SLBR-N. B) CORCEMA-ST analysis to compare theoretical and experimental STD effects of ligand **5.** For the calculation of R-NOE = 0.2 protons of sialic acids were excluded due to overlapping signals. Labels: A = GlcNAc of 3'SLn branch, B = Gal of sTa branch, C = GalNAc of sTa branch, D = Gal of 3'SLn branch.

Figure S15. Analysis of the interaction between SLBR-N and ligand **6.** A) 1D and 2D STD NMR spectra provided an epitope mapping (the remaining overlapped proton signals were not considered). B) 3D model of SLBR-N – ligand **6** complex. The interactions monitored during the MD simulation were highlighted.

Figure S16. Comparison between disialyl core 2 O-glycans. A) Superimposition of sulphated (cyan) and non-sulphated (green) disialyl core 2 O-glycans with respective 2D plots of the main representation of the complexes obtained from the cluster by MD simulations. B) 3D view of the ligands' accommodation into SLBR-N, highlighting the orientation of OH/OSO_3 at position 6 of non sulphated and sulphated disialyl core2 O-glycans, respectively, toward E285.

Figure S17. Different views of 3D models between SLBR-N and ligands **1** (gold), **2** (pink), **3** (blue), **4** (purple), **5** (green). Grey colored the protein in absence of the ligand.

Supporting References

(1) Sindrewicz, P.; Li, X.; Yates, E. A.; Turnbull, J. E.; Lian, L.-Y.; Yu, L.-G. Intrinsic tryptophan fluorescence spectroscopy reliably determines galectin-ligand interactions. *Scientific reports* **2019**, *9* (1), 11851.

(2) Gimeno, A.; Delgado, S.; Valverde, P.; Bertuzzi, S.; Berbís, M. A.; Echavarren, J.; Lacetera, A.; Martín-Santamaría, S.; Surolia, A.; Cañada, F. J. Minimizing the entropy penalty for ligand binding: lessons from the molecular recognition of the histo blood-group antigens by human galectin-3. *Angewandte Chemie International Edition* **2019**, *58* (22), 7268-7272.

(3) Forgione, R.E.; Di Carluccio, C; Guzmán-Caldentey, J; Gaglione, R; Battista, F; Chiodo, F; Manabe, Y; Arciello, A; Del Vecchio, P; Fukase, K; Molinaro, A; Martín-Santamaría, S; Crocker, PR; Marchetti, R; Silipo, A. Unveiling Molecular Recognition of Sialoglycans by Human Siglec-10. *iScience*. **2020**, 23(6), 101231.

(4) Santra, A.; Li, Y.; Ghosh, T.; Li, R.; Yu, H.; Chen, X. Regioselective one-pot multienzyme (OPME) chemoenzymatic strategies for systematic synthesis of sialyl core 2 glycans. *ACS catalysis* **2018**, *9* (1), 211-215.

(5) Xu, Z.; Deng, Y.; Zhang, Z.; Ma, W.; Li, W.; Wen, L.; Li, T. Diversity-Oriented Chemoenzymatic Synthesis of Sulfated and Nonsulfated Core 2 O-GalNAc Glycans. *The Journal of Organic Chemistry* **2021**, *86* (15), 10819-10828.

(6) Gabelica V., Livet, S., Rosu F. Optimizing Native Ion Mobility Q-TOF in Helium and Nitrogen for Very Fragile Noncovalent Structures. *J Am Soc Mass Spectrom.* **2018**, 29, 2189-2198.

(7) Du, X., Li, Y., Xia, Y.L., Ai, S.M., Liang, J., Sang, P., Ji, X.L. and Liu, S.Q. Insights into protein–ligand interactions: mechanisms, models, and methods. *International journal of molecular sciences* **2016**, 17 (2), 144.

(8) http://wiki.cara.nmr.ch/FrontPage

(9) Fernández, C.; Wider, G. TROSY in NMR studies of the structure and function of large biological macromolecules. *Current opinion in structural biology* **2003**, *13* (5), 570-580.

(10) Williamson, M. Chemical shift perturbation. *Modern Magnetic Resonance* **2018**, 995-1012.

(11) Giuntini, S.; Balducci, E.; Cerofolini, L.; Ravera, E.; Fragai, M.; Berti, F.; Luchinat, C. Characterization of the conjugation pattern in large polysaccharide–protein conjugates by NMR spectroscopy. *Angewandte Chemie* **2017**, *129* (47), 15193-15197.

(12) Mayer, M.; Meyer, B. Group epitope mapping by saturation transfer difference NMR to identify segments of a ligand in direct contact with a protein receptor. *journal of the american chemical society* **2001**, *123* (25),

(13) Meyer, B.; Peters, T. NMR spectroscopy techniques for screening and identifying ligand binding to protein receptors. *Angewandte Chemie International Edition* **2003**, *42* (8), 864-890.

(14) Szczepina, M. G.; Zheng, R. B.; Completo, G. C.; Lowary, T. L.; Pinto, B. M. STD-NMR studies suggest that two acceptor substrates for GlfT2, a bifunctional galactofuranosyltransferase required for the biosynthesis of Mycobacterium tuberculosis arabinogalactan, compete for the same binding site. *ChemBioChem* **2009**, *10* (12), 2052-2059.

(15) Lima, C. D.; Coelho, H.; Gimeno, A.; Trovão, F.; Diniz, A.; Dias, J. S.; Jiménez-Barbero, J.; Corzana, F.; Carvalho, A. L.; Cabrita, E. J. Structural Insights into the Molecular Recognition Mechanism of the Cancer and Pathogenic Epitope, LacdiNAc by Immune-Related Lectins. *Chemistry–A European Journal* **2021**, *27* (29), 7951-7958.

(16) https://glycam.org/

(17) Case, D. A.; Betz, R.; Cerutti, D.; Cheatham, T.; Darden, T.; Duke, R.; Giese, T.; Gohlke, H.; Goetz, A.; Homeyer, N. AMBER 2016 reference manual. *University of California: San Francisco, CA, USA* **2016**, 1-923.

(18) Gotz, A. W.; Williamson, M. J.; Xu, D.; Poole, D.; Le Grand, S.; Walker, R. C. Routine microsecond molecular dynamics simulations with AMBER on GPUs. 1. Generalized born. *Journal of chemical theory and computation* **2012**, *8* (5), 1542-1555.

(19) Roe, D. R.; Cheatham III, T. E. PTRAJ and CPPTRAJ: software for processing and analysis of molecular dynamics trajectory data. *Journal of chemical theory and computation* **2013**, *9* (7), 3084-3095.

(20)Jayalakshmi, V. Krishna, N. R. Complete Relaxation and Conformational Exchange Matrix (CORCEMA) Analysis of Intermolecular Saturation Transfer Effects in Reversibly Forming Ligand–Receptor Complexes. *J. Magn. Reson.* **2002**, 155 (1), 106–18.
