# Supplementary figures and images for: Molecular Insights into O-Linked Sialoglycans Recognition by the Siglec-Like SLBR-N (SLBRUB10712) of Streptococcus gordonii
Source: ACS Cent Sci. 2024 Feb 7;10(2):447–59. doi: 10.1021/acscentsci.3c01598 (PMC10906241; doi:10.1021/acscentsci.3c01598)

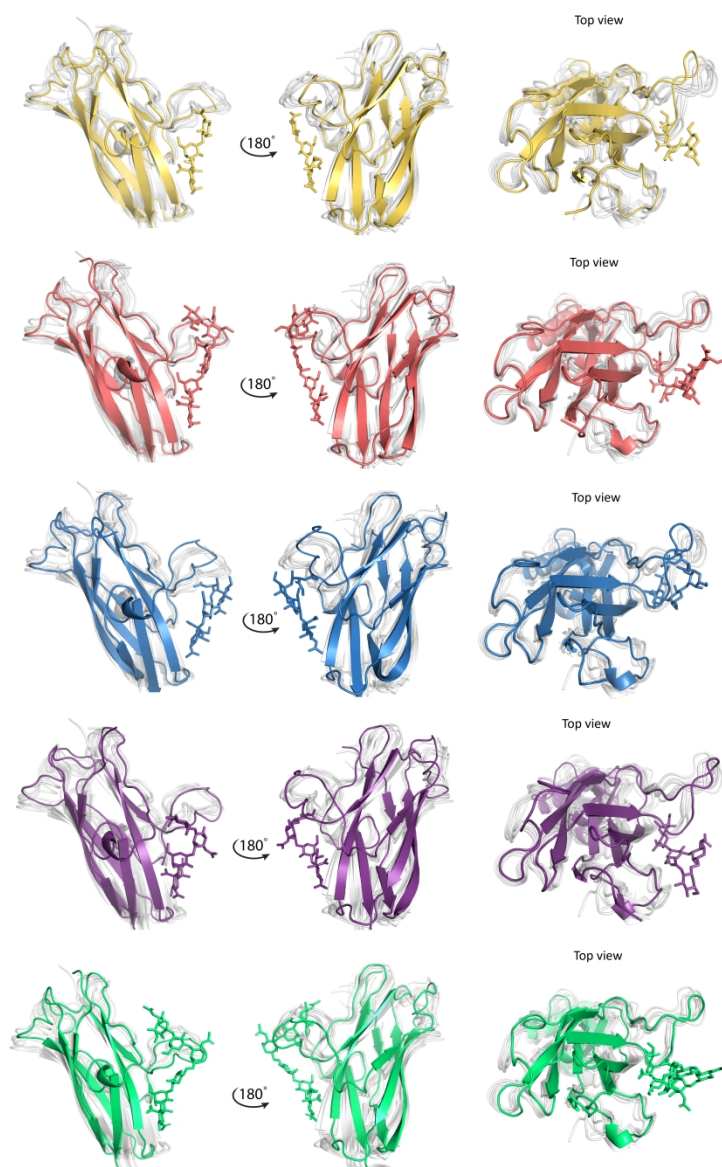

351x557mm (330 x 330 DPI)

Supplement: Supplementary file 2 — oc3c01598_si_003.pdf [file oc3c01598_si_003.pdf]
