# Supplementary material for: Molecular Insights into O-Linked Sialoglycans Recognition by the Siglec-Like SLBR-N (SLBRUB10712) of Streptococcus gordonii
Source: ACS Cent Sci. 2024 Feb 7;10(2):447–59. doi: 10.1021/acscentsci.3c01598 (PMC10906241; doi:10.1021/acscentsci.3c01598)
Supplement: Supplementary file 3 — oc3c01598_si_004.pdf [file oc3c01598_si_004.pdf]

Figure S1

A

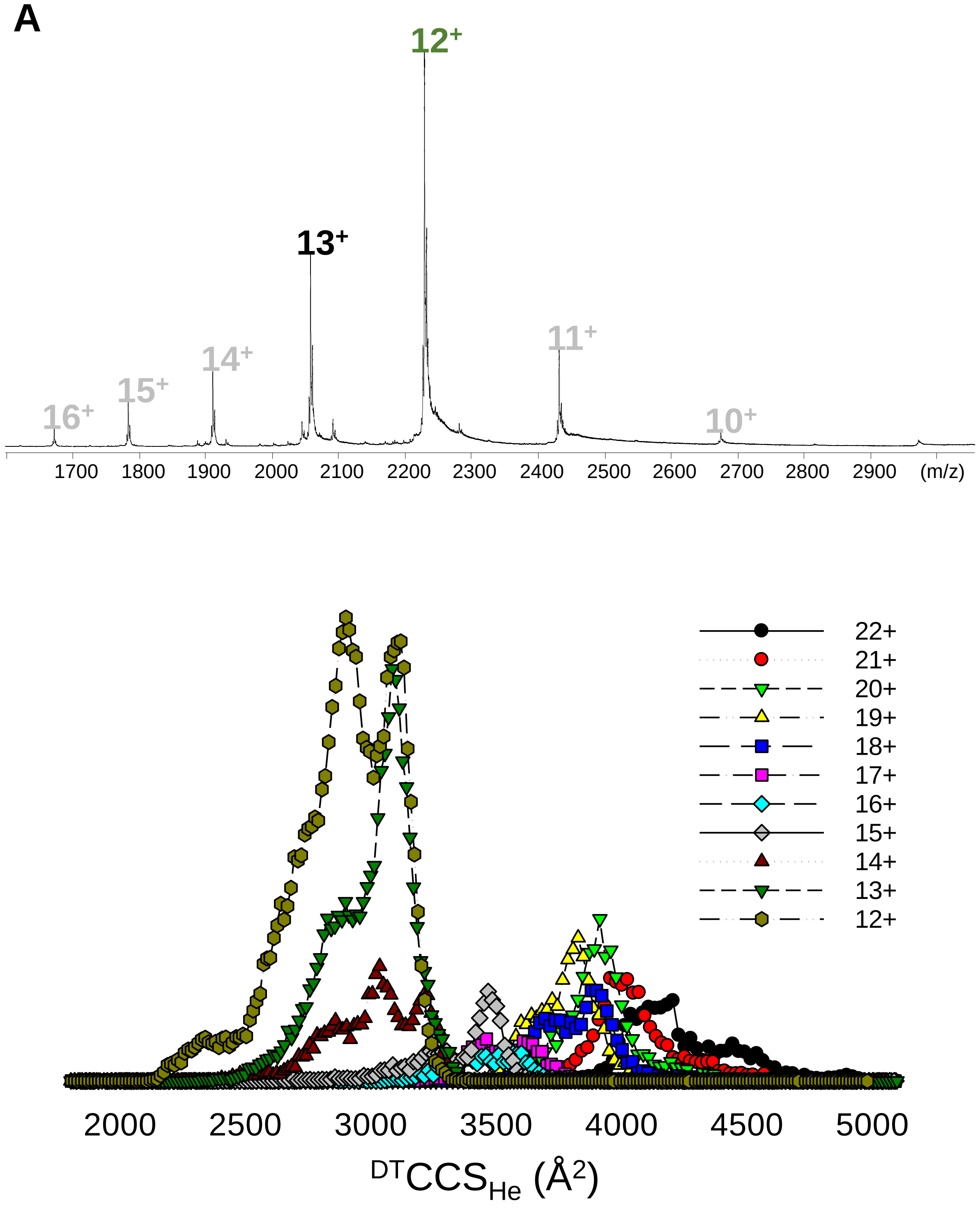

B

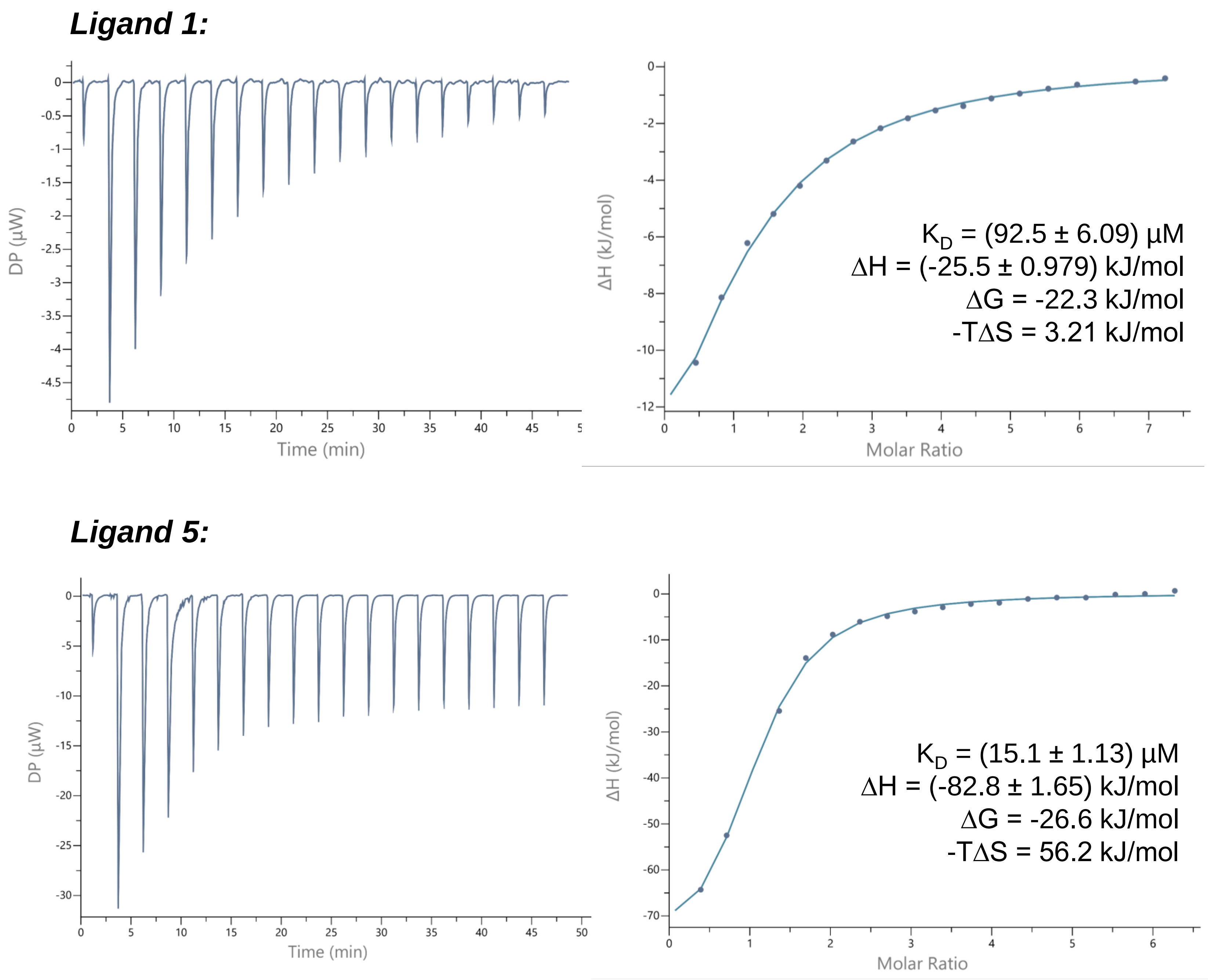

**Ligand**	**K_a (1) [M^{-1}]**	**K_a (2) [M^{-1}]**	**-log (K_a (1))**	**-log (K_a (2))**
**1**	$1.26\ 10^4$	$1.91\ 10^4$	-4.1	-4.3
**2**	$3.10\ 10^3$	-	-3.5	-
**3**	$3.08\ 10^4$	$1.28\ 10^4$	-4.5	-4.1
**4**	$8.00\ 10^3$	-	-3.9	-
**5**	$9.44\ 10^4$	$1.75\ 10^4$	-5.0	-4.2
**6**	$1.38\ 10^4$	$1.13\ 10^4$	-4.1	-4.1

Figure S2

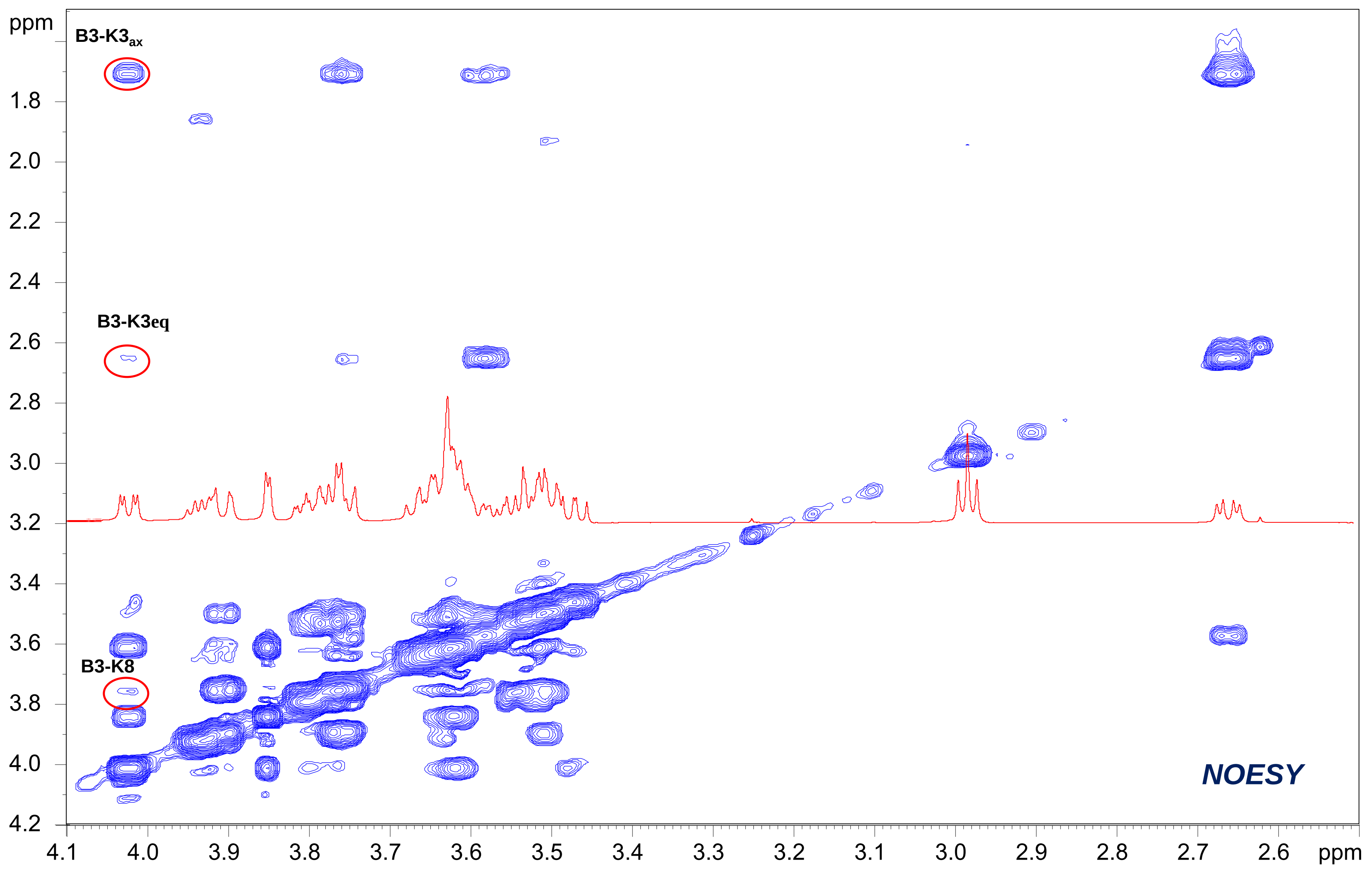

Figure S3

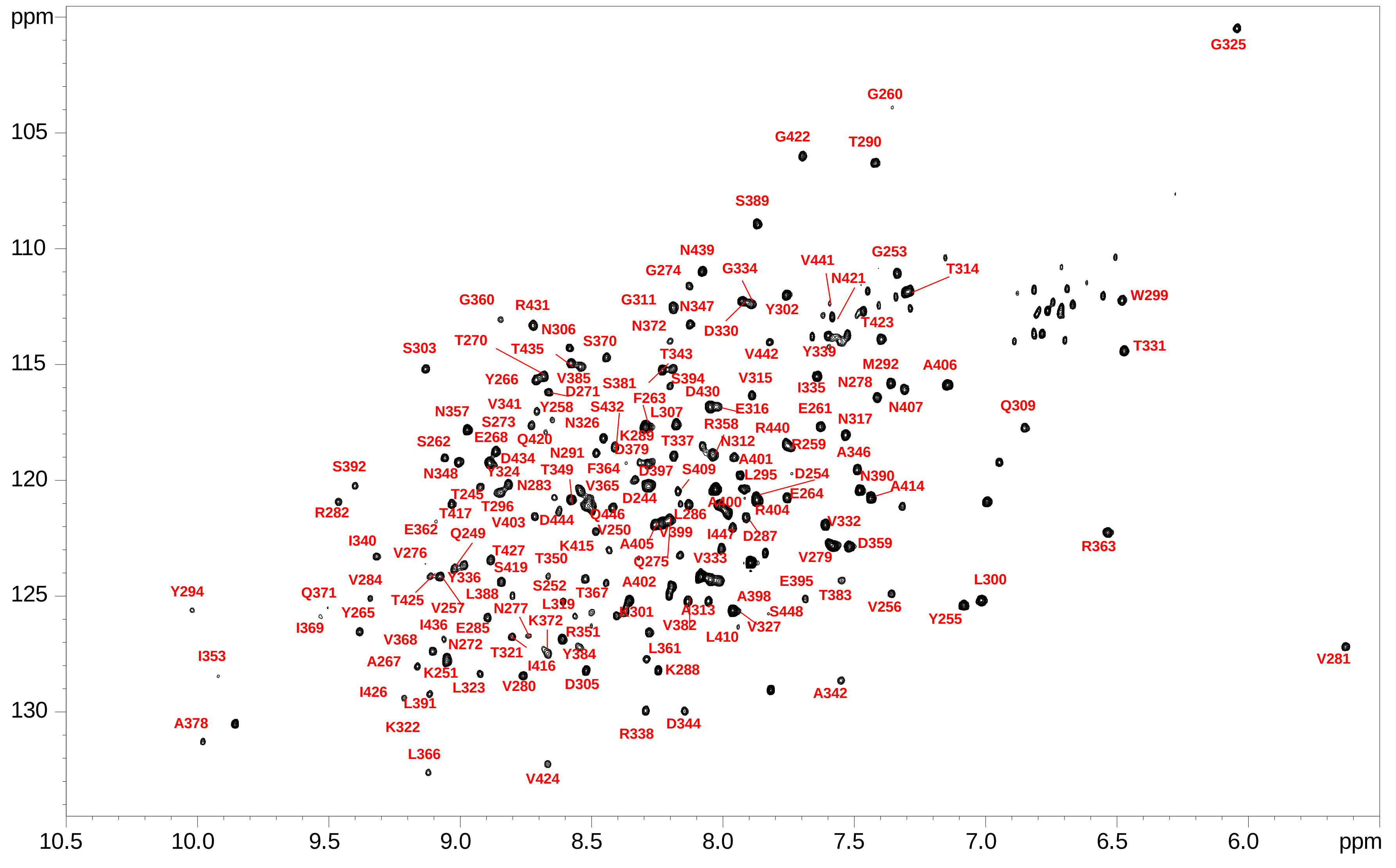

Figure S4

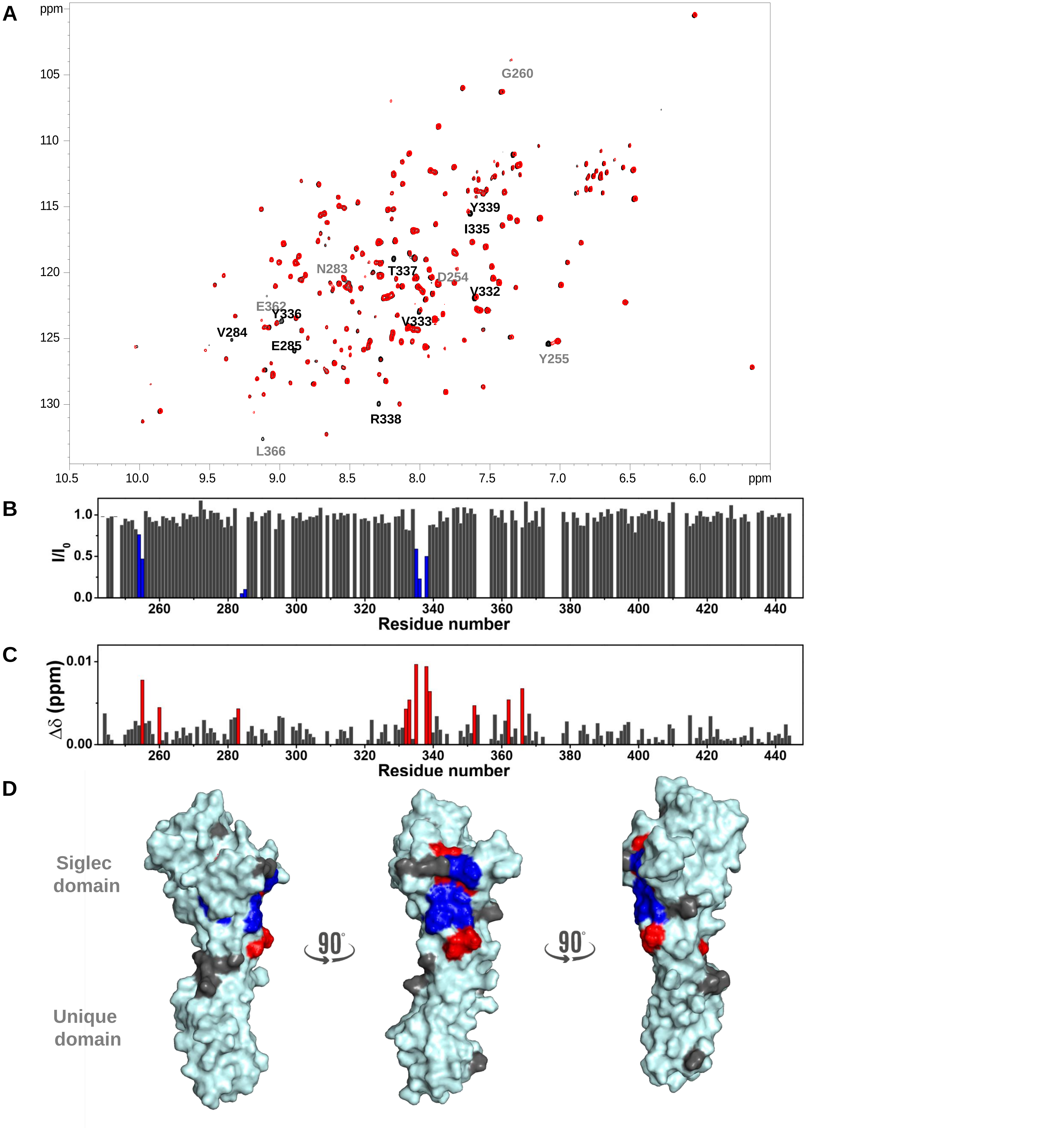
A
ppm
105
110
115
120
125
130
10.5
10.0
9.5
9.0
8.5
8.0
7.5
7.0
6.5
6.0
ppm
G260
Y339
I335
N283
T337
D254
V332
E362
Y336
V284
V333
E285
Y255
R338
L366
B
I/I0
1.0
0.5
0.0
260
280
300
320
340
360
380
400
420
440
Residue number
C
Δδ (ppm)
0.01
0.00
260
280
300
320
340
360
380
400
420
440
Residue number
D
Siglec domain
Unique domain
90°
90°

Figure S5

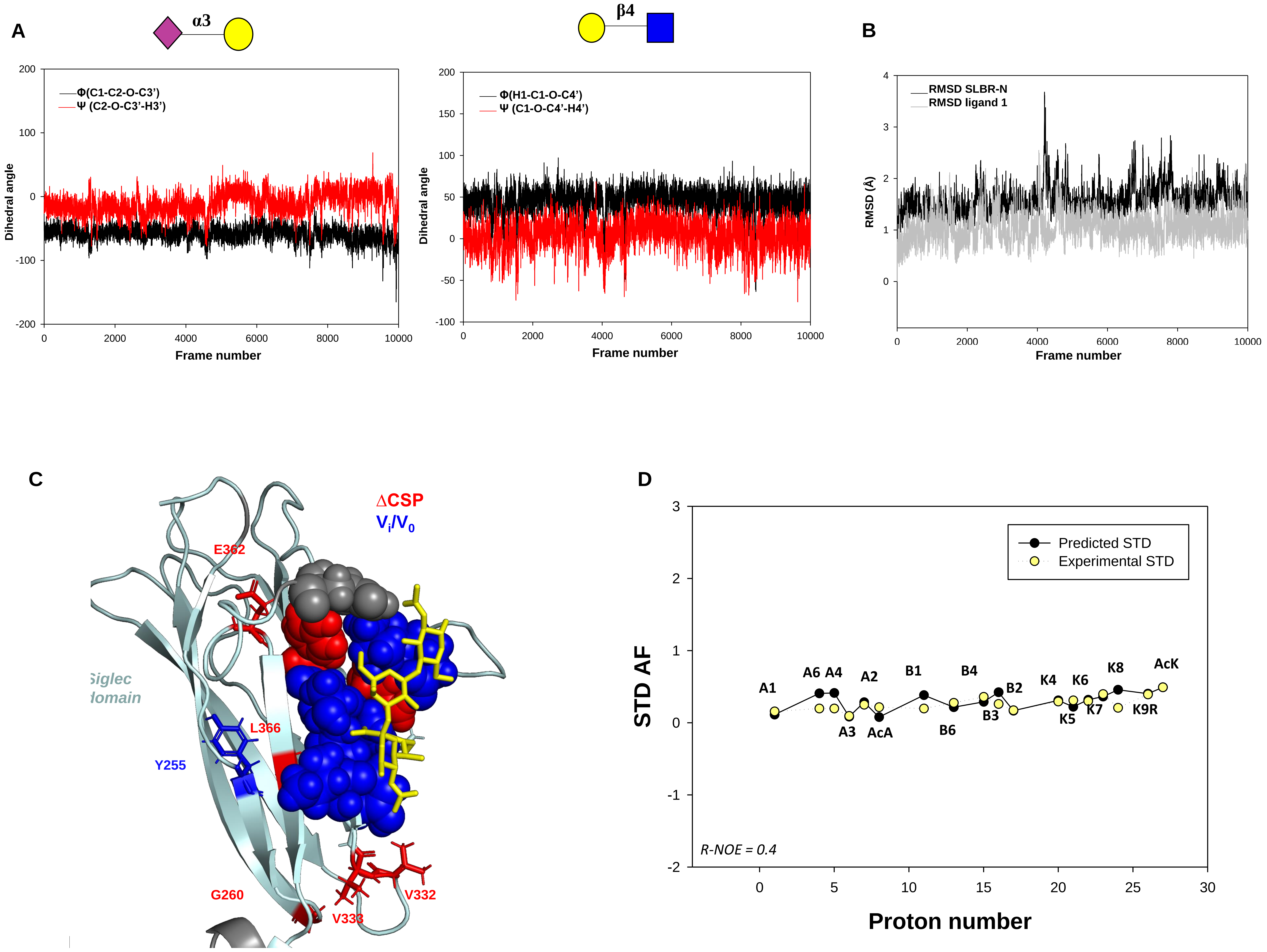

A
α3
β4
200
100
0
-100
-200
Φ(C1-C2-O-C3')
Ψ (C2-O-C3'-H3')
Dihedral angle
0
2000
4000
6000
8000
10000
Frame number
150
50
-50
Φ(H1-C1-O-C4')
Ψ (C1-O-C4'-H4')
B
RMSD SLBR-N
RMSD ligand 1
RMSD (Å)
C
ΔCSP
V_i/V_0
E362
L366
Y255
G260
V333
V332
Siglec domain
D
Predicted STD
Experimental STD
STD AF
Proton number
R-NOE = 0.4
A1
A6
A4
A2
A3
AcA
B1
B6
B4
B3
B2
K4
K5
K6
K7
K8
K9R
AcK

Figure S6

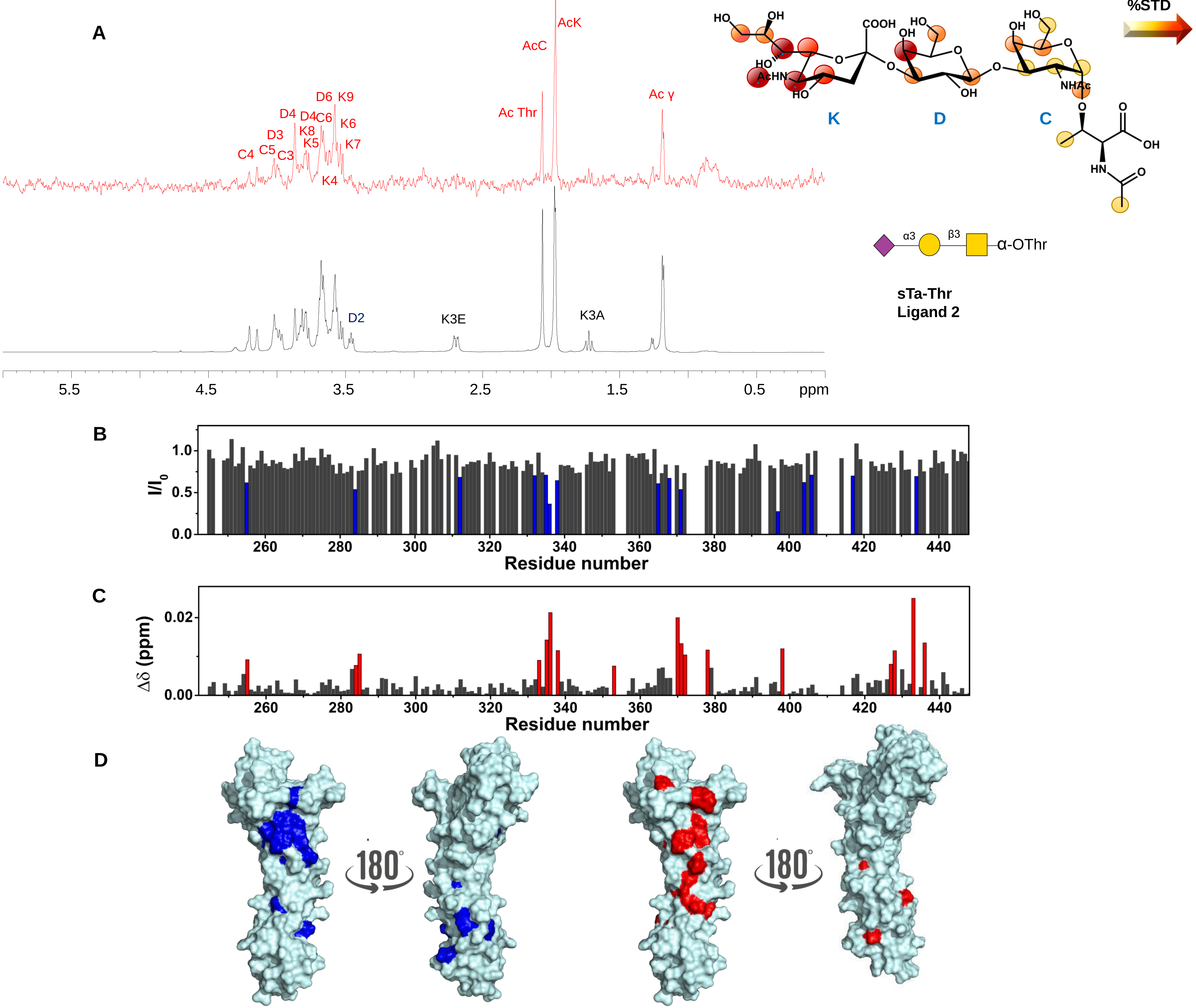
A
AcK
AcC
D6 K9
D4
D4C6
K6
D3
K8
K5
K7
C4 C5 C3
K4
Ac Thr
Ac γ
D2
K3E
K3A
5.5
4.5
3.5
2.5
1.5
0.5
ppm
%STD
K
D
C
α3
β3
α-OThr
sTa-Thr
Ligand 2
B
I/I_0
Residue number
C
Δδ (ppm)
Residue number
D
180°
180°

Figure S7

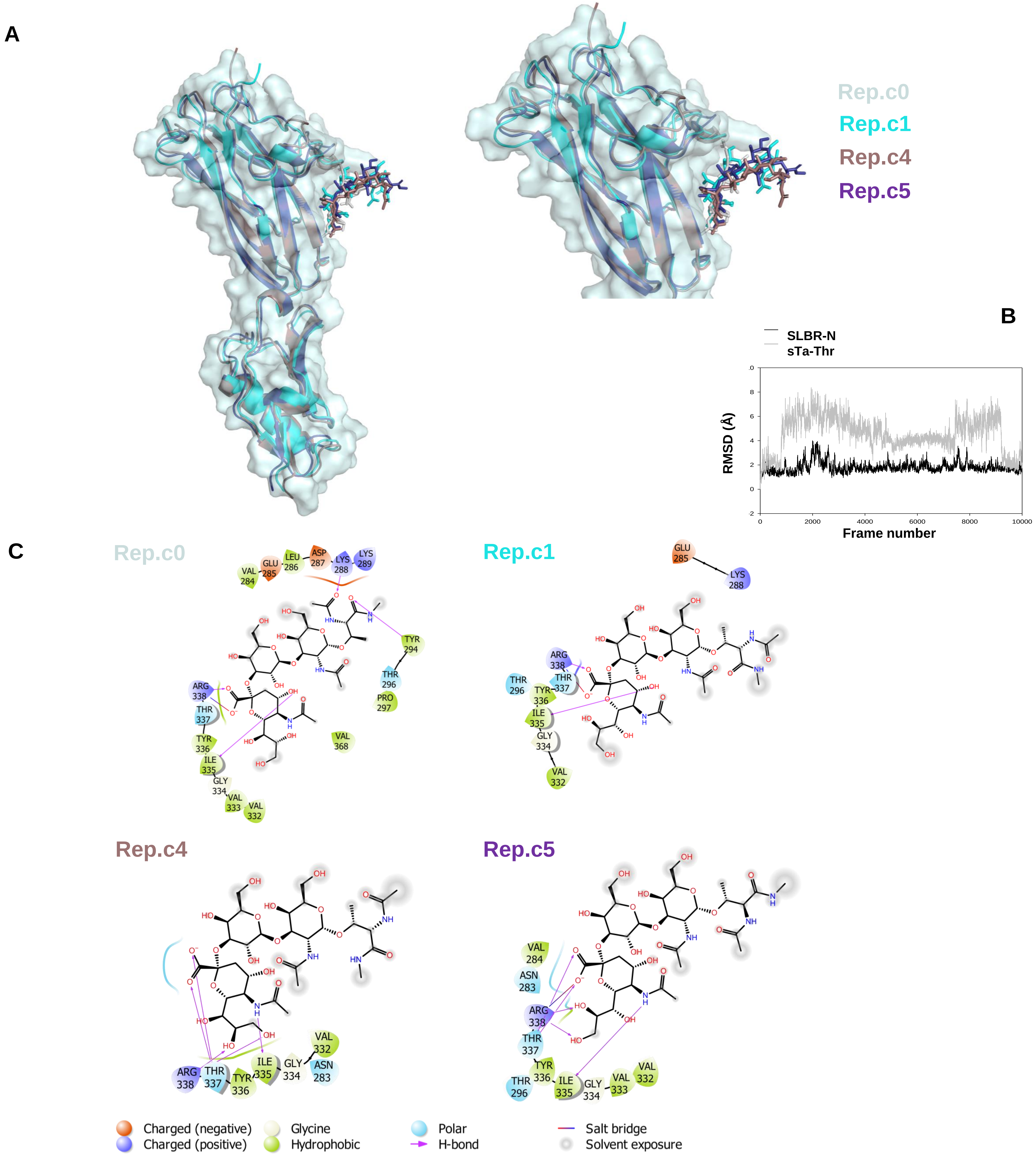
A
Rep.c0
Rep.c1
Rep.c4
Rep.c5
B
SLBR-N
sTa-Thr
RMSD (Å)
Frame number
C
Rep.c0
Rep.c1
Rep.c4
Rep.c5
Charged (negative)
Charged (positive)
Glycine
Hydrophobic
Polar
H-bond
Salt bridge
Solvent exposure

Figure S8

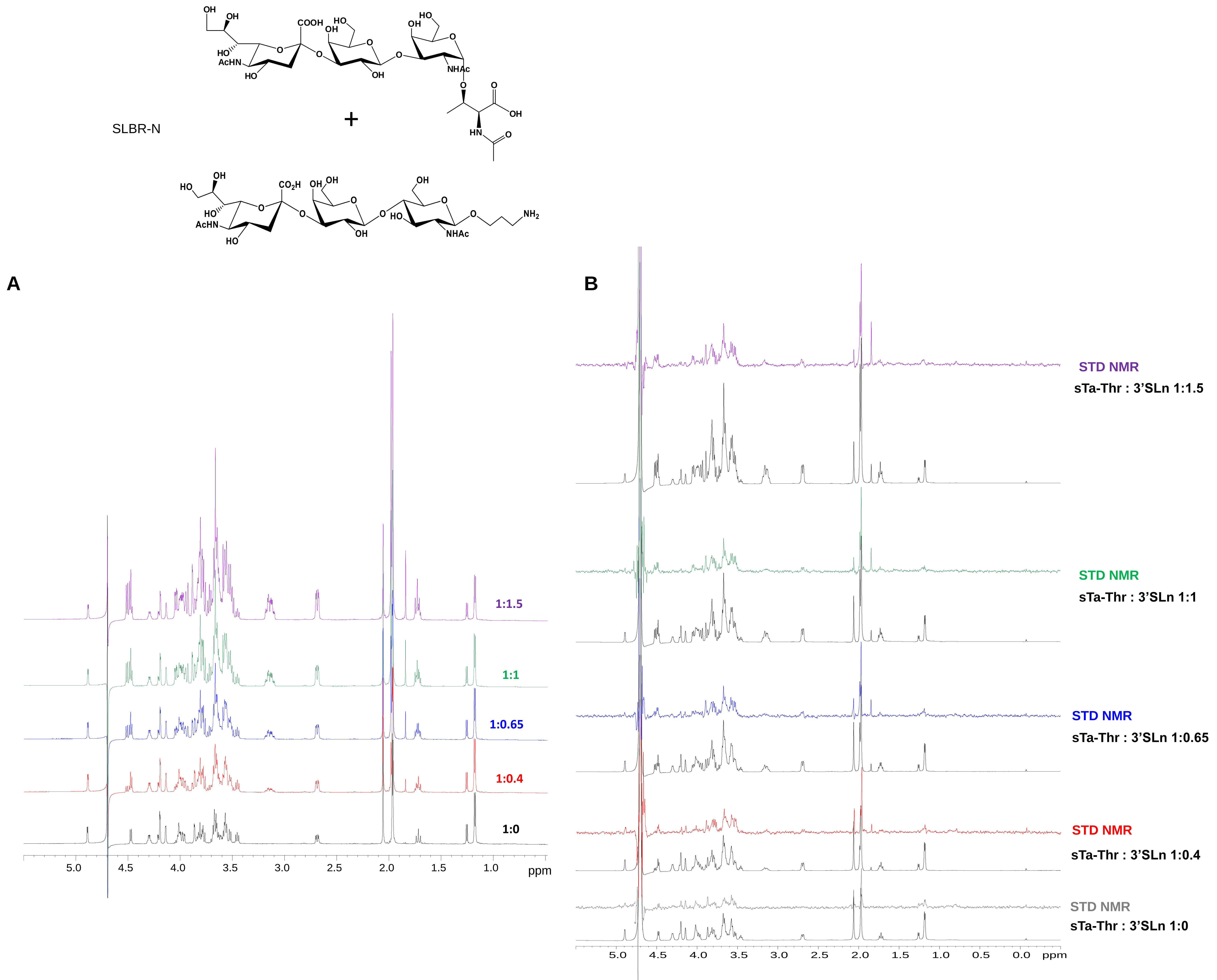

SLBR-N
+
A
1:1.5
1:1
1:0.65
1:0.4
1:0
ppm
B
STD NMR
sTa-Thr : 3'SLn 1:1.5
STD NMR
sTa-Thr : 3'SLn 1:1
STD NMR
sTa-Thr : 3'SLn 1:0.65
STD NMR
sTa-Thr : 3'SLn 1:0.4
STD NMR
sTa-Thr : 3'SLn 1:0
ppm

Figure S9

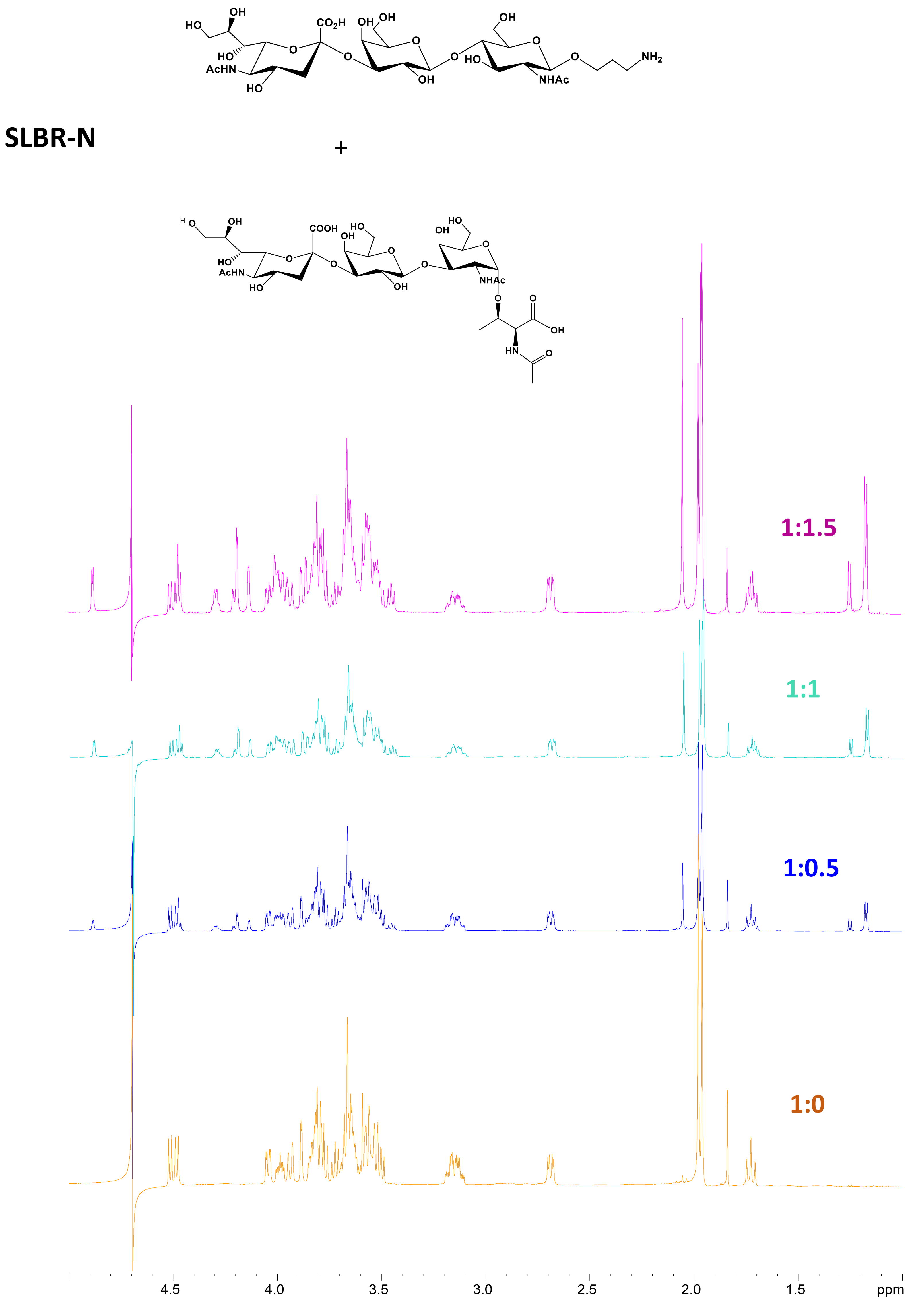

Figure S10

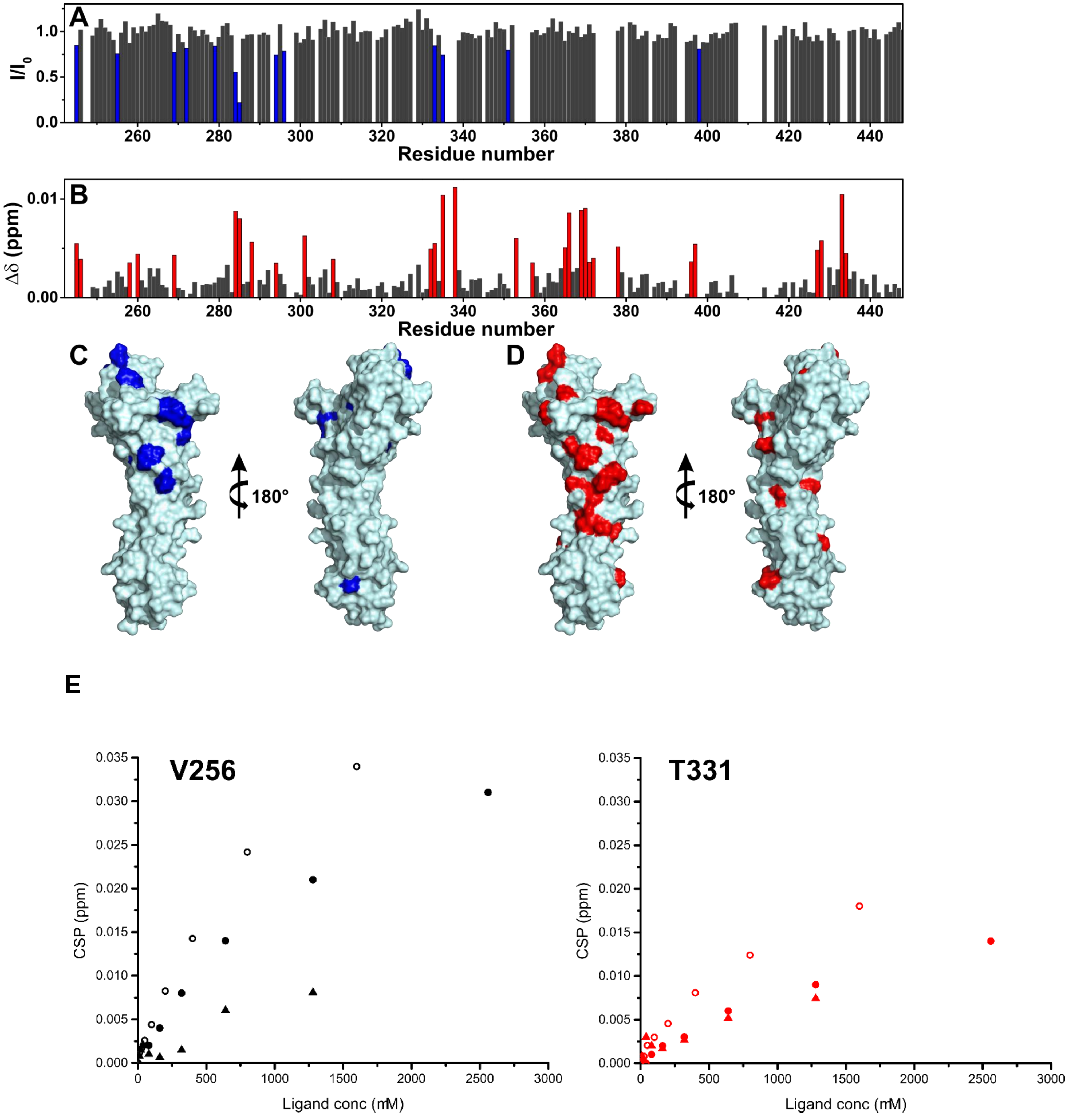

A
B
C
D
E
I/I0
Δδ (ppm)
Residue number
180°
V256
T331
CSP (ppm)
Ligand conc (mM)

Figure S11

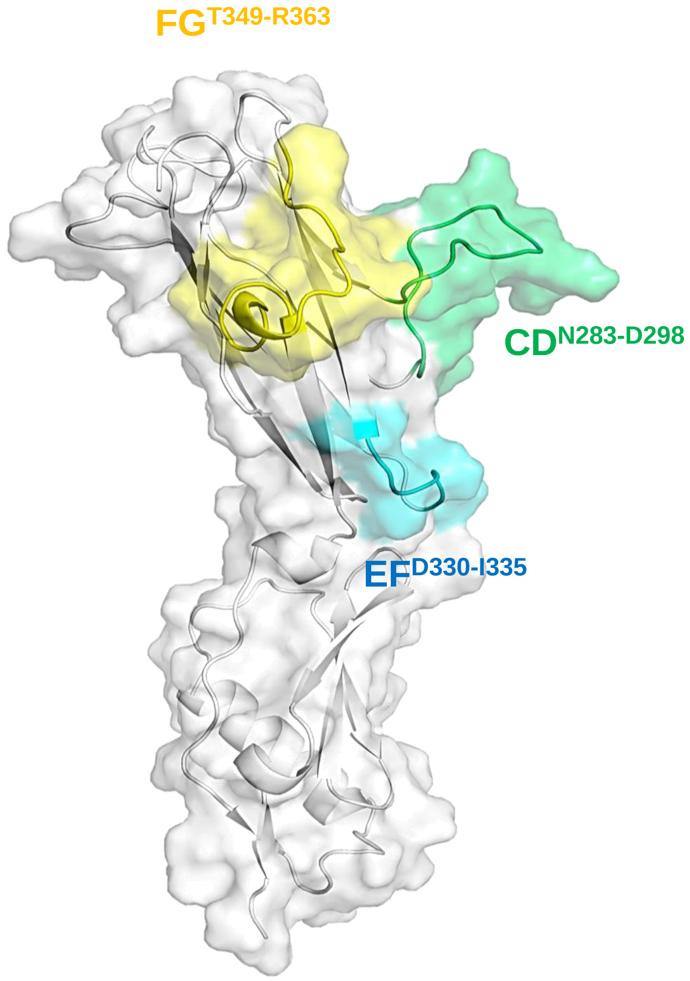
FG T349-R363
CD N283-D298
EF D330-I335

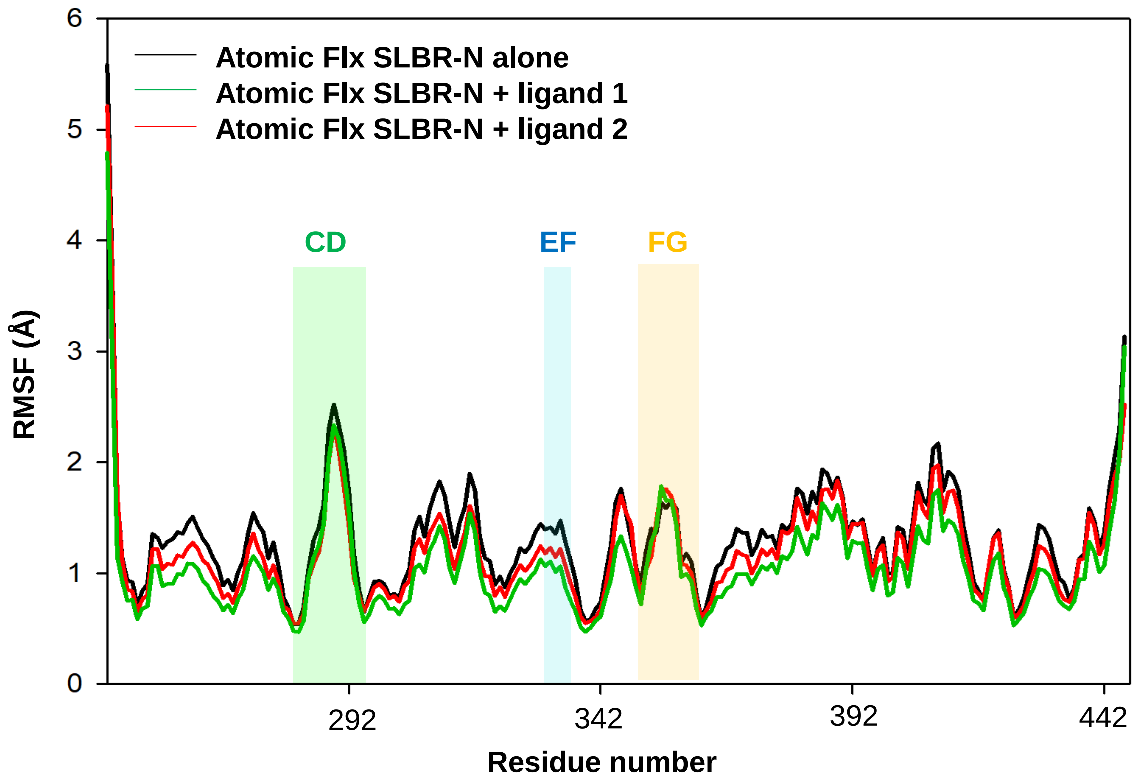
Atomic Flx SLBR-N alone
Atomic Flx SLBR-N + ligand 1
Atomic Flx SLBR-N + ligand 2
CD
EF
FG
RMSF (Å)
Residue number
0
1
2
3
4
5
6
292
342
392
442

Figure S12

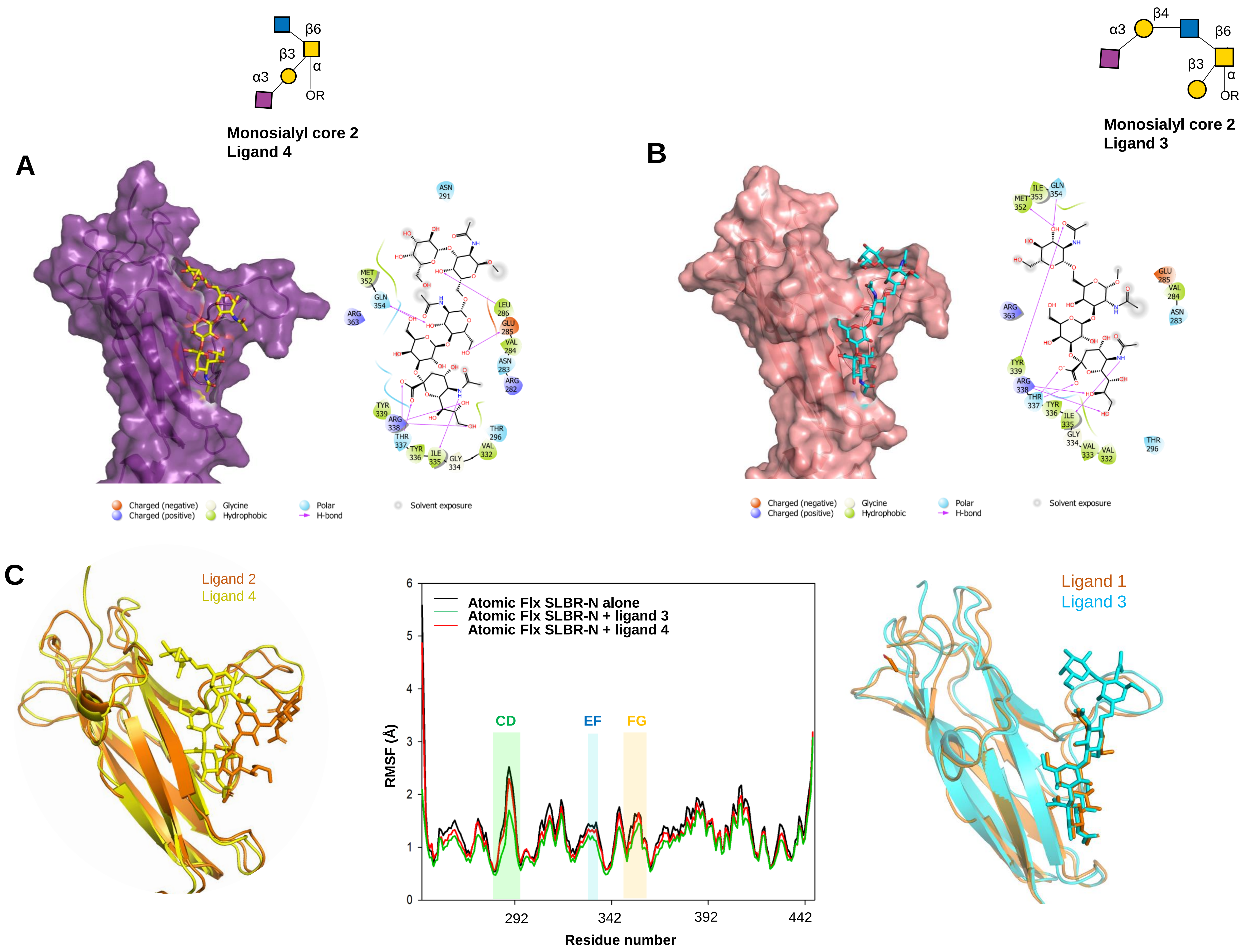

A
B
C
β6
β3
α
α3
OR
Monosialyl core 2
Ligand 4
β4
α3
β6
β3
α
OR
Monosialyl core 2
Ligand 3
Charged (negative)
Charged (positive)
Glycine
Hydrophobic
Polar
H-bond
Solvent exposure
Ligand 2
Ligand 4
Atomic Flx SLBR-N alone
Atomic Flx SLBR-N + ligand 3
Atomic Flx SLBR-N + ligand 4
CD
EF
FG
RMSF (Å)
Residue number
292
342
392
442
Ligand 1
Ligand 3

Figure S13

Free state

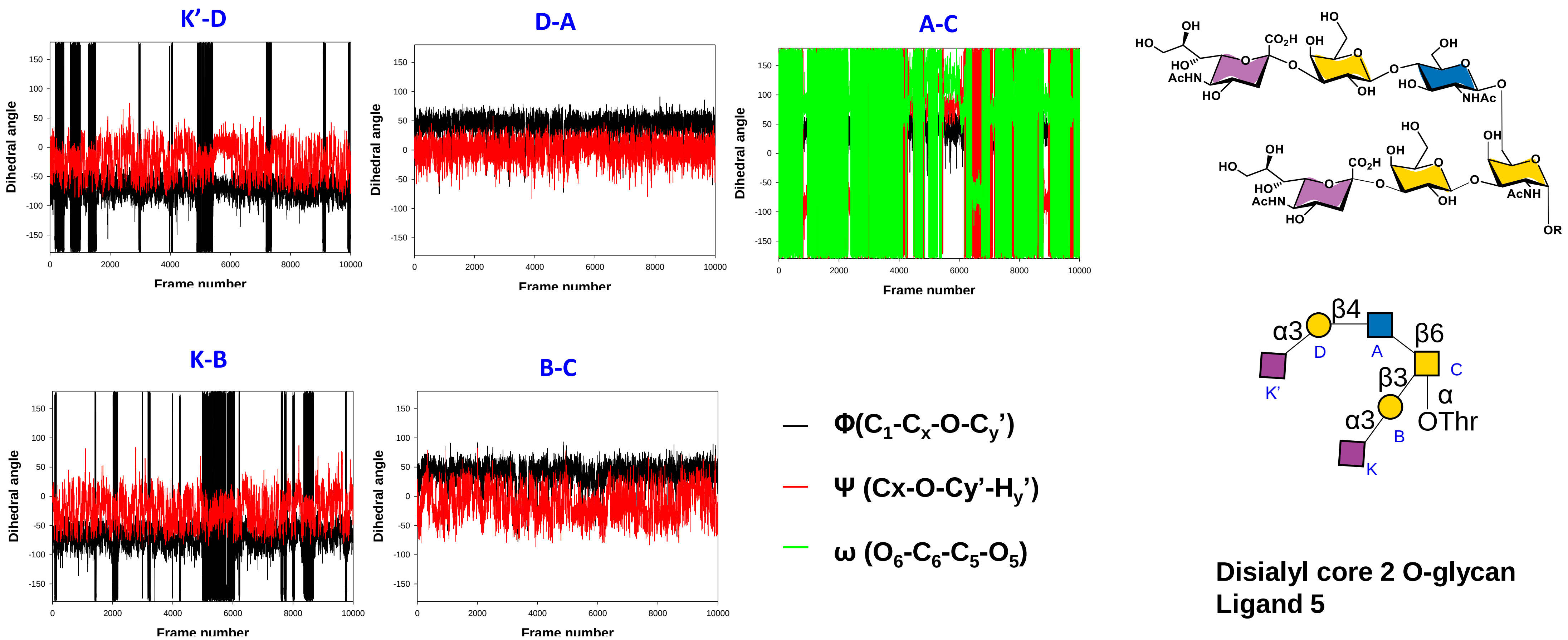

Figure S14

A

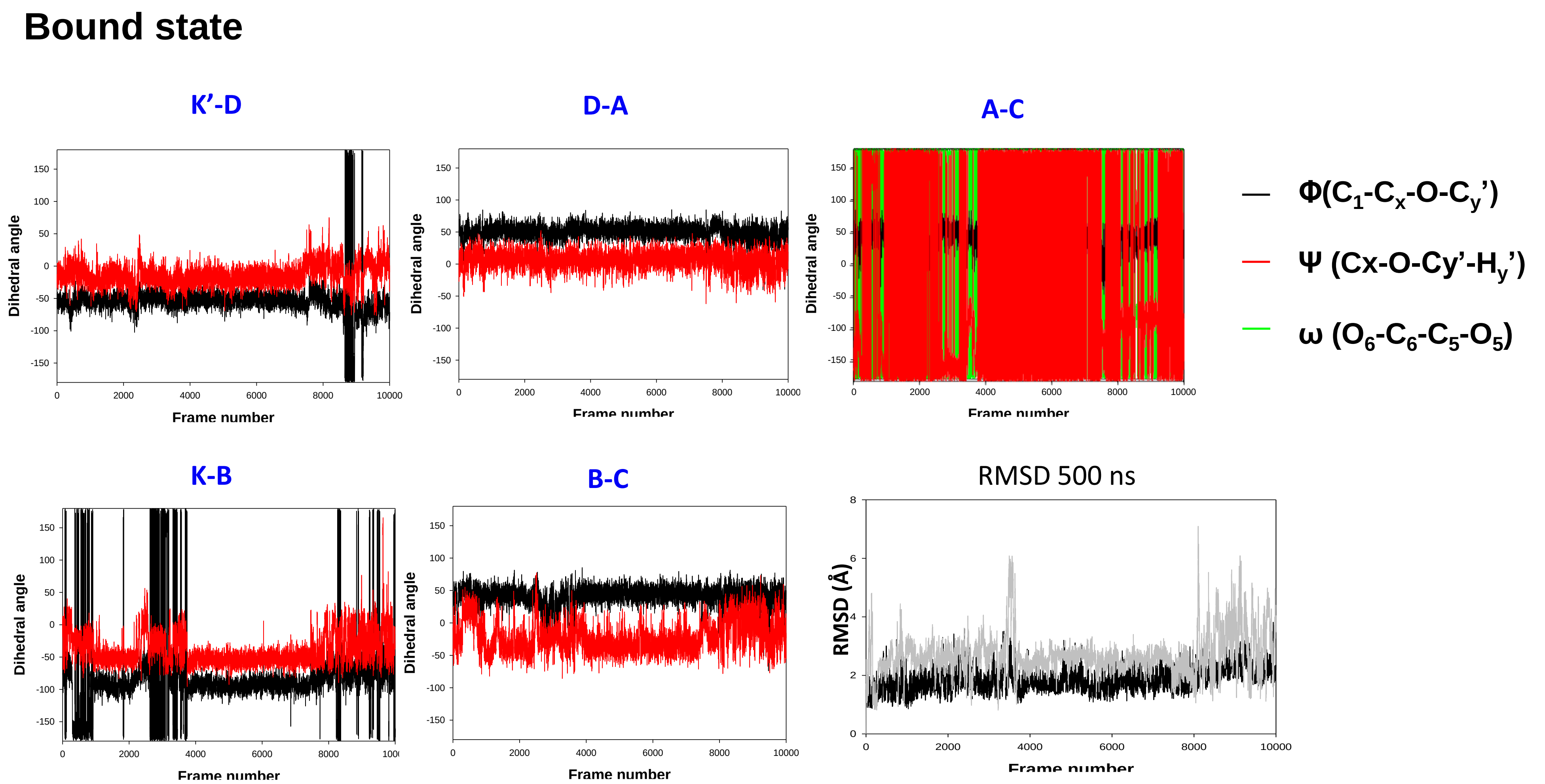

B

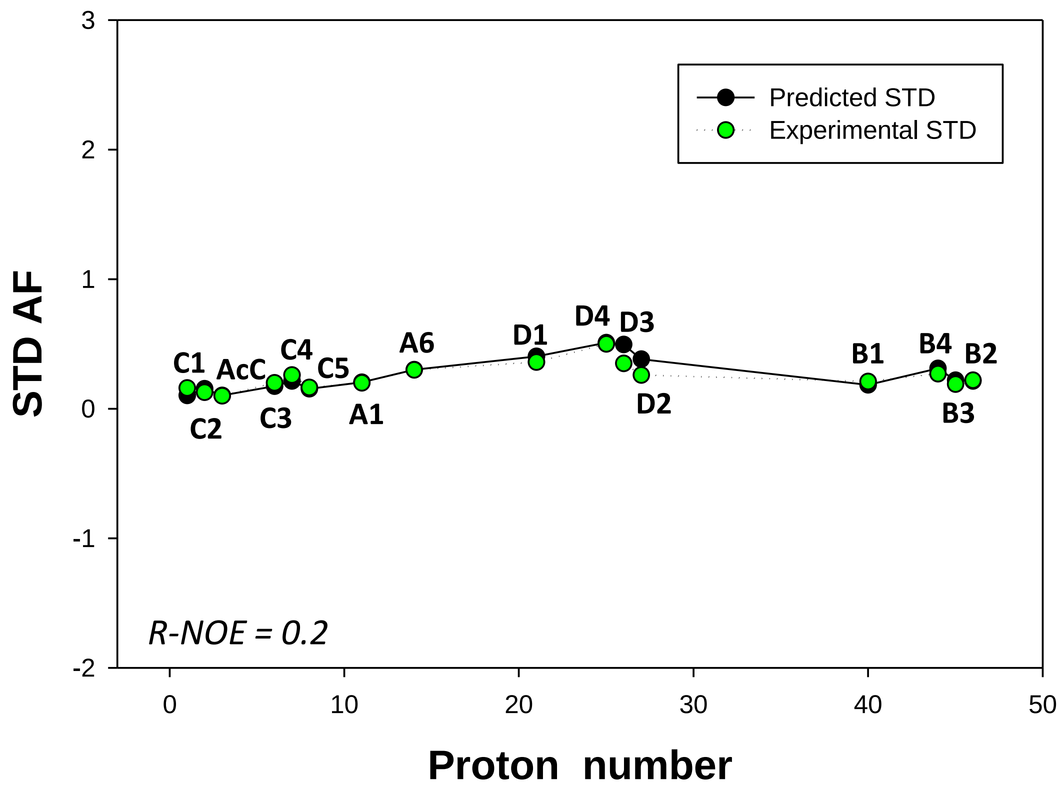

Figure S15

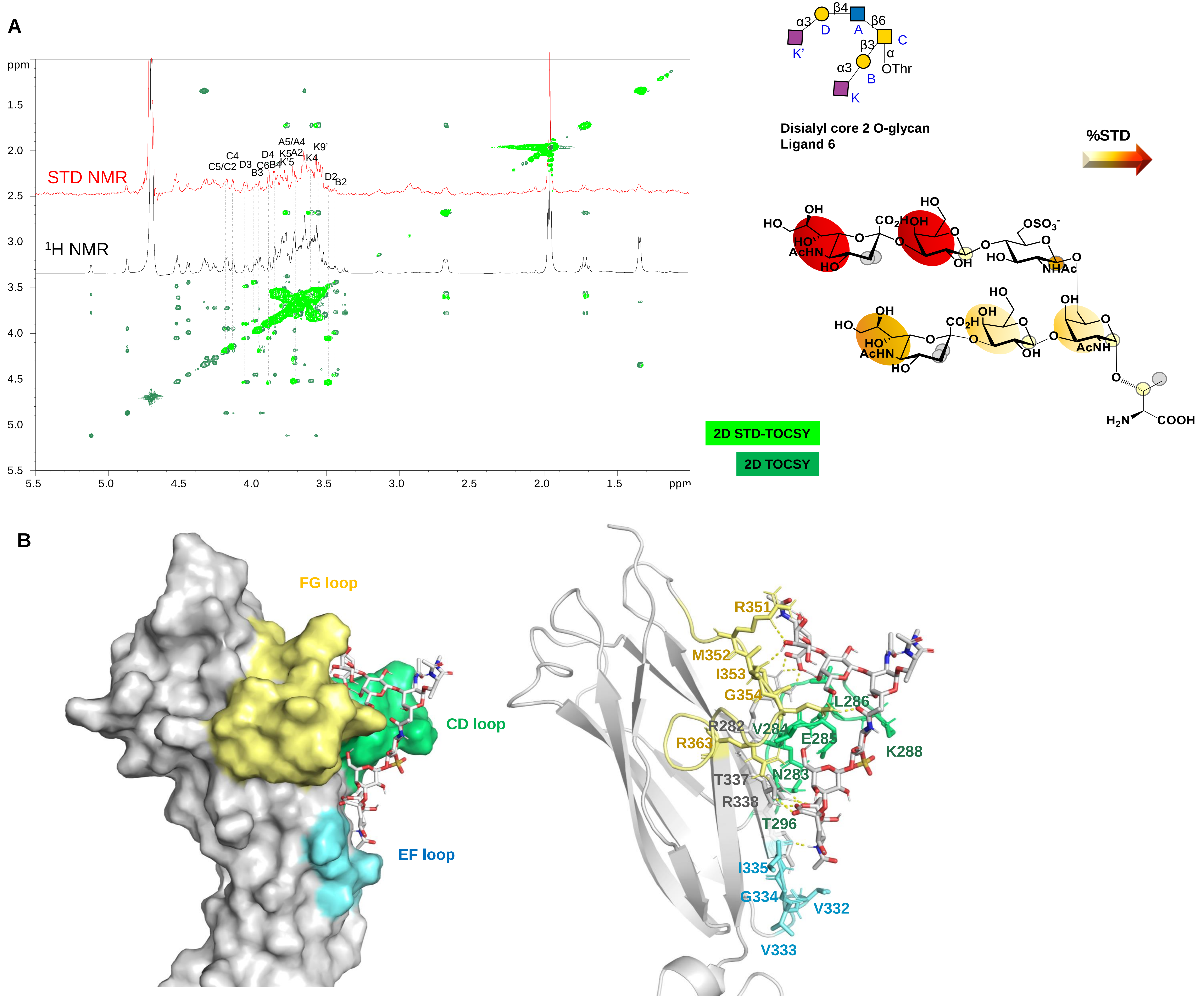
A
STD NMR
^{1}H NMR
C4
C5/C2
D3
D4
C6B4
K'5
A5/A4
K5A2
K9'
K4
B3
D2
B2
ppm
2D STD-TOCSY
2D TOCSY
6S
β4
β6
α3
β3
α
OThr
K'
D
A
C
B
K
Disialyl core 2 O-glycan
Ligand 6
%STD
B
FG loop
CD loop
EF loop
R351
M352
I353
G354
L286
R282
V284
E285
R363
K288
T337
N283
R338
T296
I335
G334
V332
V333

Figure S16

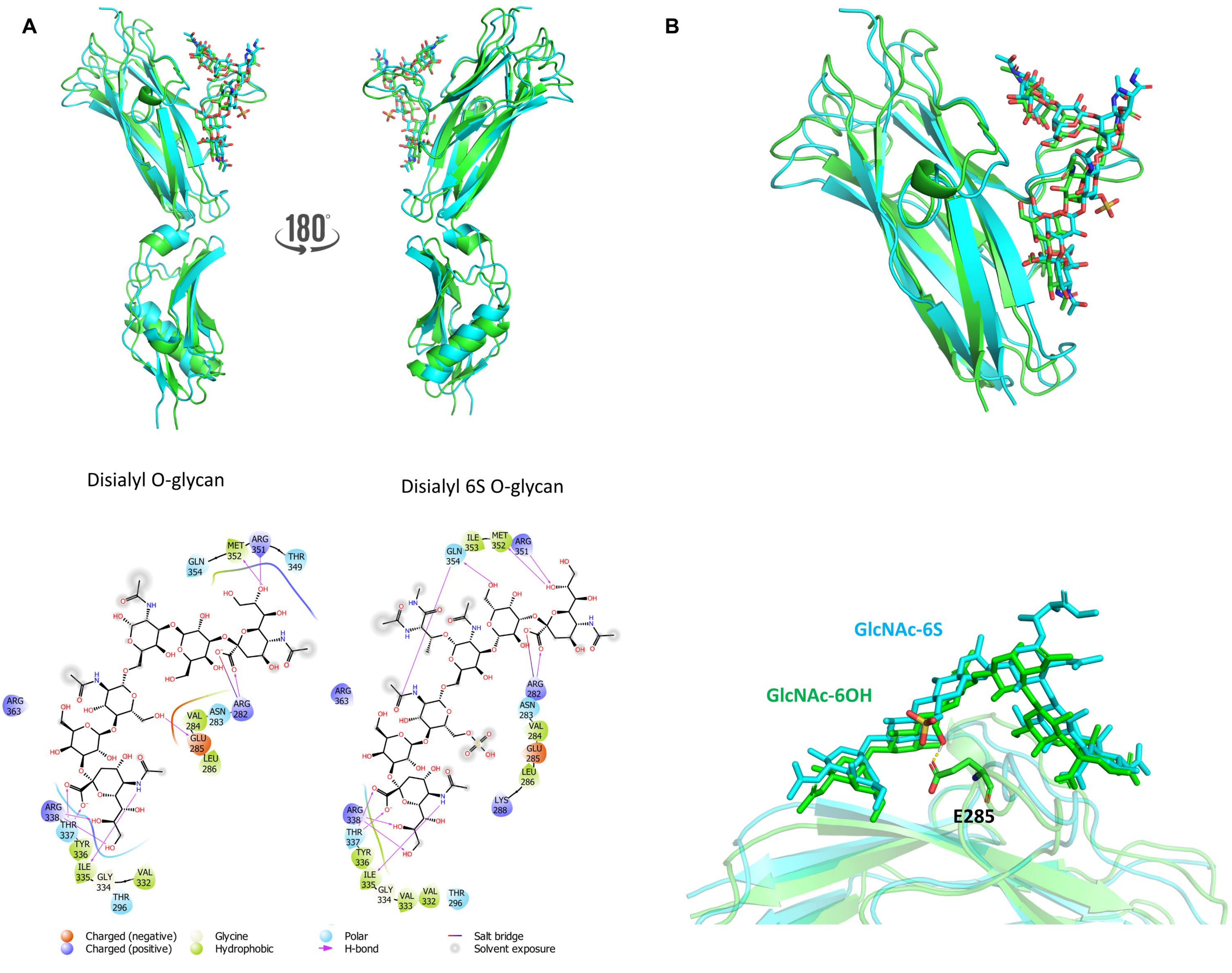

A
B
180°
Disialyl O-glycan
Disialyl 6S O-glycan
GlcNAc-6S
GlcNAc-6OH
E285
Charged (negative)
Charged (positive)
Glycine
Hydrophobic
Polar
H-bond
Salt bridge
Solvent exposure
